# Adipose Tissue Dysfunction and Altered Systemic Amino Acid Metabolism Are Associated with Non-Alcoholic Fatty Liver Disease

**DOI:** 10.1371/journal.pone.0138889

**Published:** 2015-10-06

**Authors:** Sulin Cheng, Petri Wiklund, Reija Autio, Ronald Borra, Xiaowei Ojanen, Leiting Xu, Timo Törmäkangas, Markku Alen

**Affiliations:** 1 Exercise Health and Technology Centre, Shanghai Jiao Tong University, Shanghai, China; 2 Department of Health Sciences, University of Jyvaskyla, Jyvaskyla, Finland; 3 Department of Signal Processing, Tampere University of Technology, Tampere, Finland; 4 Department of Diagnostic Radiology, University of Turku and Turku University Hospital, Turku, Finland; 5 Department of Applied Physics, University of Eastern Finland, Kuopio, Finland; 6 Medical School, Ningbo University, Ningbo, China; 7 Department of Medical Rehabilitation, Oulu University Hospital, Oulu, Finland; 8 School of Health Sciences, University of Tampere, Tampere, Finland; University of Basque Country, SPAIN

## Abstract

**Background:**

Fatty liver is a major cause of obesity-related morbidity and mortality. The aim of this study was to identify early metabolic alterations associated with liver fat accumulation in 50- to 55-year-old men (n = 49) and women (n = 52) with and without NAFLD.

**Methods:**

Hepatic fat content was measured using proton magnetic resonance spectroscopy (^1^H MRS). Serum samples were analyzed using a nuclear magnetic resonance (NMR) metabolomics platform. Global gene expression profiles of adipose tissues and skeletal muscle were analyzed using Affymetrix microarrays and quantitative PCR. Muscle protein expression was analyzed by Western blot.

**Results:**

Increased branched-chain amino acid (BCAA), aromatic amino acid (AAA) and orosomucoid were associated with liver fat accumulation already in its early stage, independent of sex, obesity or insulin resistance (p<0.05 for all). Significant down-regulation of BCAA catabolism and fatty acid and energy metabolism was observed in the adipose tissue of the NAFLD group (p<0.001for all), whereas no aberrant gene expression in the skeletal muscle was found. Reduced BCAA catabolic activity was inversely associated with serum BCAA and liver fat content (p<0.05 for all).

**Conclusions:**

Liver fat accumulation, already in its early stage, is associated with increased serum branched-chain and aromatic amino acids. The observed associations of decreased BCAA catabolism activity, mitochondrial energy metabolism and serum BCAA concentration with liver fat content suggest that adipose tissue dysfunction may have a key role in the systemic nature of NAFLD pathogenesis.

## Introduction

The prevalence of non-alcoholic fatty liver (NAFLD) is on the rise, currently affecting up to 30% of the adult population and an increasing number of children in the developed countries [1]. In the early phase, NAFLD is asymptomatic, benign and often reversible, but if not controlled, it may progress to non-alcoholic steatohepatitis (NASH), cirrhosis and ultimately liver failure [2]. Therefore, metabolic perturbations contributing to the development of the disease should be identified before clinical manifestations occur. The metabolic abnormalities related to fatty liver are reflected in the level of circulating metabolites. Thus, comprehensive metabolic profiling holds potential for identifying specific disease-related patterns and non-invasive biomarkers [3]

Metabolic profiling studies have commonly noted increased free fatty acids and other lipid species in the plasma or serum of subjects with NAFLD [4]. In particular, acylcarnitines, lysophosphatidylcholines and triacylglycerol with low carbon number and double-bond content, have emerged as auspicious liver fat biomarkers [5,6]. Recent studies have also demonstrated increased circulating branched-chain amino acids (BCAAs) and their metabolic intermediates in subjects with NAFLD [7–9] and NASH[9,10] compared to healthy controls, but the underlying mechanisms of these associations remain to be established. These findings indicate that metabolic profiling can provide important information about etiology of the development and progression of NAFLD. However, the influence of extra-hepatic tissues on hepatic steatosis is incompletely understood.

Since NAFLD is closely associated with obesity, insulin resistance and type 2 diabetes[11–13], it is suspected that adipose tissue and skeletal muscle may play an important role in the development of NAFLD. Indeed, growing evidence indicates that adipose tissue dysfunction [14] and increased secretion of adipokines [15] are implicated in the systemic nature of NAFLD pathogenesis. Experimental studies have demonstrated that skeletal muscle insulin resistance promotes liver fat accumulation by altering the distribution pattern of postprandial energy storage [16]. Skeletal muscle has also been shown to modulate adipose tissue metabolism [17]. The newly discovered myokine, irisin, which has been proposed to convey the inter-organ signaling between skeletal muscle and adipose tissue, has recently been associated with NAFLD [18,19]. Furthermore, both skeletal muscle and adipose tissue are significant regulators of systemic amino acid metabolism, as most of the catabolic activity of BCAAs resides in these tissues [20]. Therefore, tissue-specific alterations in BCAA metabolism may contribute to the elevated levels of circulating BCAAs associated with fatty liver [21,22].

The aim of our study was to identify early systemic metabolic alterations associated with liver fat accumulation in healthy middle-aged men and women with and without NAFLD. In addition, we studied global gene expression profiles of adipose tissues and skeletal muscle, the purpose being to describe the early changes in the metabolic pathways that accompany liver fat accumulation and relate these to the serum metabolite profiles and associated clinically relevant factors.

## Materials and Methods

### Study participants

This article is a part of the Calex family study (n = 282 families), which has been described elsewhere [23,24]. For the purpose of this report, a subgroup of families (n = 74), comprising 222 individuals (daughter, mother and father) with no history of liver, pancreas or heart disease, or of heavy drinking, were contacted by letters for an additional study aimed at identifying the early metabolic alterations associated with liver fat accumulation. A total of 184 individuals responded to our invitation, of whom 163 (53 fathers, 53 mothers and 57 daughters) attended the laboratory tests. For this report, all the daughters were excluded owing to the low number with NAFLD (n = 5), leaving only the mothers and fathers (n = 106). Valid measurements of liver fat were unavailable for two men. In addition, two men reported recent alcohol consumption of >21 drinks on average per week and one woman reported >14 drinks on average per week. These individuals were therefore excluded. Hence, the final numbers of participants were 49 men and 52 women. Thirty (n = 30) participants had NAFLD, as defined by the cut-off LFC value of >5.56% [25]. The remainder, with a LFC value of <5.56%, were assigned to the healthy control group (n = 71). Of these 101 subjects, 32 (11 with NAFLD and 21 healthy controls) agreed to donate subcutaneous and skeletal muscle (vastus lateralis) biopsies.

Health history and current status was checked by the study physician. No major liver (cancer, hepatitis), pancreas (type I/II diabetes) or cardiac diseases were found. However, five men (3 healthy controls and 2 NAFLD) and two women (1 healthy control and 1 NAFLD) were using statins, and thirteen men (6 healthy controls and 7 NAFLD) and four women (3 controls and 1 NAFLD) were using hypertension medication. In addition, one man and three women were using thyroxine for hypothyreosis. All the other subjects were clinically euthyreotic. Twenty-two women were in early post menopause (15 healthy controls and 7 NAFLD), but there was no difference between the two groups in menopausal age, and none of the women were on hormonal replacement therapy. Including or excluding these women did not influence the results, and hence they were included in the final analysis. The study protocol was approved by the ethics committee of the Central Finland Health Care District. A written informed consent was obtained from all participants.

### Background information, liver fat content, abdominal fat mass and myocellular lipid assessment

Body height, weight and body mass index (BMI) were assessed and the results reported elsewhere [23,26]. Dietary intake of total energy and energy-yielding nutrients were assessed from three-day food records and analyzed using Micro-Nutrica software developed by the Social Insurance Institution of Finland and updated with data for new foodstuffs by the study nutritionist [27]. Leisure time physical activity (PA), including walking, jogging, running, gym fitness, ball games, swimming, etc., expressed as hours/week and times/week, was evaluated using a validated self-administered physical activity questionnaire, as described previously [28].

Whole body fat mass (FM) was assessed by Dual-energy X-ray absorptiometry (DXA Prodigy, GE Lunar Corp., Madison, WI USA). In this study, two repeated measurements of FM showed a coefficient of variation (CV) of 2.2% [23].

Liver and abdominal regions were scanned using a 1.5 Tesla MR scanner (GE Signa CV/i, General Electric Healthcare, Waukesha, WI, USA). LFC was assessed by ^1^HMRS with a PRESS sequence and was analysed using the Linear Combination of Model spectra software which is generally considered to be the gold standard for in-vivo spectroscopy analysis [29,30].

Abdominal adipose tissue compartments (subcutaneous = SAT, visceral = VAT, retroperitoneal = RAT) were quantified from a single slice image at the level of the L2-L3 intervertebral disc using OsiriX software (OsiriX Foundation, Geneva, Switzerland). The results were converted into tissue fat mass in kg taking slice thickness into account and assuming an adipose tissue density of 0.9196 g/ml [31].

Muscle intra-myocellular lipid (IMCL) and extra-myocellular lipid (EMCL) from the tibialis anterior muscle were measured using a similar ^1^H MRS method with a surface coil placed over the middle part of the muscle[32]. In order to obtain maximal IMCL and EMCL separation, the tibialis anterior muscle was aligned as closely as possible with the direction of the magnetic field and the voxel was placed parallel to the muscle fibers[32].

### Biochemical assessments

Blood samples were collected in the morning between 7:00 and 9:00 am after overnight fasting. Among the women with regular menses, the samples were collected between 2 and 5 days after menstruation onset. Plasma glucose, serum alkaline phosphatase (ALP), alanine aminotransferase (ALT), aspartate aminotransferase (AST), γ-glutamyltransferase (GGT) and non-esterified fatty acids (NEFA) were assessed by a KONELAB 20XTi analyzer (Thermo Fischer Scientific inc. Waltham, MA, USA). Plasma insulin was assessed by chemiluminescent immunoassay (Diagnostic Products Corporation, Los Angeles). The intra- and inter-assay CVs were 3.4% and 2.0% for glucose, 11% and 3.4% for insulin, and 7.4% and 8.4% for NEFA. The HOMA-IR index (homeostatic model assessment of insulin resistance) was calculated as (fasting glucose x fasting insulin/22.5). Serum leptin was assessed using human leptin (ELISA; Diagnostic Systems Laboratories, Inc., Webster, TX). Total adiponectin was measured by an enzyme immunoassay method using the Quantikine human total adiponectin/Acrp30 immunoassay (R&D Systems, Minneapolis, MN). The inter- and intra-assay coefficients of variation (CVs) were 2.2% and 2.7% for leptin, and 3.3% and 4.3% for adiponectin. Serum high-sensitivity C-reactive protein (hsCRP) was assessed using an ELISA DuoSet (R&D Systems and Diagnostic Systems Laboratories, Inc). The intra- and inter-assay CVs were 4.6% and 6.9%.

### Serum NMR spectroscopy

All serum samples were analyzed using a high-throughput serum NMR metabolomics platform; the experimental protocols, including sample preparation and NMR spectroscopy, have been described in detail elsewhere [33,34]. Altogether 130 metabolites were assessed.

### Subcutaneous adipose tissue biopsies

Subcutaneous adipose tissue biopsies were obtained from 16 men and 16 women under local anesthesia after an overnight fast. A region 5 cm lateral from the umbilicus either to the left side or right side was sterilized. A small intracutaneous injection was made, and 2 ml of a local anesthetic agent (lidocaine) was injected. After 5 min, anesthesia was confirmed. The skin was then sterilized again and 10ml of 0.9% sodium-chloride was aspirated using a 16 G x 40 mm needle fitted to a 50-mL syringe. Approximately two-thirds of the length of the needle was inserted into the subcutaneous fat, and 5 ml of 0.9% sodium chloride was injected. The needle piston was then pulled back maximally and released until it was locked by a stopper, thereby creating a vacuum. Tissue resistance was created by gripping the abdominal skin with one hand while the other hand rotated the needle (back and forth) throughout the tissue (by a back and forth motion). Once the tissue had been aspirated by the syringe, the needle was withdrawn, and the piston removed. The adipose tissue samples were washed with saline solution, and were immediately frozen in liquid nitrogen and stored at—80°C.

### Skeletal muscle biopsies

Vastus lateralis biopsies were taken from 16 men and 16 women under local anesthesia after an overnight fast. Biopsies were taken from the vastus lateralis dx muscle with a 5-mm Bergström biopsy needle, midway between the patella and greater trochanter. The location and optimum depth for the muscle biopsy were confirmed by ultrasound imaging. The skin covering the identified location was sterilized and 4 ml of local anesthetic agent (lidocaine) was injected into the subdermal tissue. A cooling pack was then applied to the location. After 10 minutes, anesthesia was confirmed, the skin was sterilized again and a small stab incision made with a surgical scalpel. Next, the biopsy needle, attached to a syringe, was introduced perpendicularly into the incision. The piston was then pulled back maximally, creating a vacuum, and the sample was obtained. After this, pressure was applied to the incision site to induce hemostasis. The muscle sample was cleaned of any visible connective and adipose tissue, as well as blood, and was frozen immediately in liquid nitrogen (−180°C) and stored at −80°C.

### RNA extraction

Total RNA was extracted from the biopsies using the FastPrep system (MP Biomedicals, France) and a RNeasy Lipid Tissue Mini Kit (QIAGEN, Gaithersburg, MD, USA) according to manufacturer’s instructions. Total RNA was digested on column with a RNase-free DNase set (QIAGEN) during RNA isolation. The quality of the total RNA was studied using a 2100 Bioanalyzer (Agilent, Santa Clara, CA, USA) and Experion Automated Electrophoresis Station (BioRad, Hercules, CA, USA). The total RNA was amplified and processed using a Gene Chip 3´ IVT Express Kit (Affymetrix, Santa Clara, CA, USA) and hybridized on Affymetrix Human Genome U219 Array Plates. The samples of this study have been submitted to ArrayExpress.

### Protein extraction and Western blot from skeletal muscle biopsies

The muscle biopsies were homogenized in ice-cold lysis buffer [20 mM HEPES (pH 7.4), 1 mM EDTA, 5 mM EGTA, 10 mM Mg2Cl, 100 mM β-glycerophosphate, 1 mM Na3VO4, 1 mM DTT, 1% Triton-X-100], supplemented with protease and phosphatase inhibitors inhibitors (Sigma Aldrich, St Louis, MO, USA).

Fifty to sixty micrograms of muscle lysate samples were separated by SDS-Page using 4–20% gradient gels on a Criterion electrophoresis cell (Bio-Rad Laboratories, Richmond, CA). Proteins were transferred to nitrocellulose membranes at 300-mA constant current on ice at 4°C. Membranes were blocked in TBS containing 5% nonfat dry milk for 1 hour at room temperature (RT), and then probed overnight at 4°C with commercially available primary antibodies. All antibodies were diluted 1:1000 (except anti-GAPDH (housekeeping, which was diluted 1:40,000) in TBS containing 5% nonfat dry milk. Membranes were then washed with TBS containing 0.1% Tween-20 (TBS-T) followed by 1 hour incubation with the secondary antibody. Blots were visualized and quantified using an Odyssey CLX Infrared Imager of (Li-COR Biosciences) and the manufacturer's software. When re-probing was needed, the membranes were incubated in 0.2 M NaOH for 10 min at RT, washed with TBS and re-probed with appropriate antibodies. All samples were run in the same gel to minimize variability and the quantitative results for each protein were normalized to GAPDH.

### Transcriptomics analysis

Analysis of the transcriptomics data have been reported earlier [1]. Briefly, the gene values of the expression measurements were analyzed by using the Robust Multiarray Averaging (RMA) algorithm, as implemented in the R package affy. We ran the differentially expressed genes (DEG) analysis with the LimmaR package utilizing linear modeling and empirical Bayes methods. Raw p-values were adjusted using the Benjamini and Hochberg multiple adjustment method.

### Quantitative PCR

The results of the adipose tissue microarray analyses were confirmed by qPCR as described earlier [1]. Briefly, qPCR was performed on MMP9 and VAV1 from the same RNA samples. A High Capacity cDNA Synthesis Kit (Applied Biosystems, Foster City, CA, USA) was used to reverse transcribe 230 ng of RNA. Real-time PCR analysis was performed using iQ SYBR Supermix and CFX96™ Real-time PCR Detection System (Bio-Rad Laboratories, Richmond, CA, USA).

The primer sequences were as follows:

MMP9 sense: 50-GAGTGGCAGGGGGAAGATGC-30, and antisense 50-CCTCAGGG

CACTGCAGGATG-30

VAV1 sense: 50-AGCAGTGGGAAGCACAAAGTATT-30, and antisense 50-GTCAC

GGGCGCAGAAGTC-30

GAPDH sense: 50-CCACCCATGGCAAATTCC-30 and antisense: 50-TGGGATTTCCATTGATGACAA- 30

Relative expression levels for MMP9 and VAV1 were calculated with the DDCt method and normalized to the expression of GAPDH. The fold changes in each gene between the groups were similar to those detected in the microarray analysis (data not shown).

### Gene enrichment analysis

The enriched Gene Ontology (GO) terms or Kyoto Encyclopedia of Genes and Genomes (KEGG) pathways for a given gene set were calculated by utilizing the R packages GOStats [2]. In the enrichment analysis, all the human ENSEMBL genes were used as a background gene group. Categories with a p-value lower than 0.05 are considered significantly enriched. We detected the genes whose expression was related to liver fat content by utilizing the following two criteria: 1) the DEG analysis with an adjusted p-value of <0.05 identified genes that were differentially expressed in the healthy control and NAFLD groups, and 2) the genes had a fold change of > 1 in the NAFLD group compared to the healthy controls. The mean-centroid value representing the “activity” of the regulated part of the pathway was computed by normalizing the expression levels of all the genes in the subset to a mean of zero across all individuals. Mean centroids have previously been shown to correlate with various metabolic and physiologic parameters [22,35], and may therefore be used to assess gene expression patterns that are associated with metabolic diseases. Correlation analyses with liver fat and serum metabolites were performed as described the next section.

### Statistical methods

Continuous data were checked for normality by Shapiro-Wilk’s test before each analysis using PASW statistics version 21 (IBM Corporation, USA). If data were not normally distributed, their natural logarithms were used. Since the data were from a family study, shared environmental (household) similarity was controlled for in the analysis. The linear mixed model was used to compare levels of the outcome variables between the NAFLD and healthy control groups. Contrast tests were used in mixed models to assess the effect of gender while controlling for dependency among family members with random effects.

The metabolomics data were clustered utilizing a hierarchical clustering algorithm which has been reported elsewhere [34]. The results are expressed as means with standard deviation (SD). P-values were adjusted to control for the false discovery rate (FDR) using the method of Benjamini and Hochberg when comparing metabolites between the healthy control and NAFLD groups [36]. To identify relevant metabolites associated with LFC, we used principal component analysis to reduce a large number of correlated variables to fewer uncorrelated factors. Metabolite factor scores were calculated for each individual based on the constructed scoring coefficients. The principal component score is a transformation on the values of the metabolites, which can be considered as a weighted measure of the variability shared by the variables. Fasting levels of amino acids, fatty acids, phospholipids, glycoproteins, ketone bodies, and glycolysis and gluconeogenesis intermediates were included in the principal component analysis. Mean metabolite factor levels were compared between the NAFLD and healthy control groups. To exclude the possibility of misclassification, we divided the participants into quintiles based on liver fat content and compared their mean metabolite factor levels adjusting for HOMA-IR, BMI and visceral fat mass. Pearson correlation analyses were performed to determine the relationship between the gene pathways and clinical characteristics. Statistical significance was set at p< 0.05.

## Results

### Anthropometry, fat depots, lifestyle factors and conventional serum biomarkers

The mean liver fat content in the NAFLD and healthy control groups were 13.6% vs. 1.9% (p<0.001). No group by gender interaction was observed in liver fat content. The NAFLD participants were heavier and had higher BMI, total, visceral and retroperitoneal FM and IMCL compared to the healthy controls (p≤0.005 for all, **Table 1**). The NAFLD participants had higher fasting glucose, insulin, HOMA-IR, triglycerides, hsCRP, NEFA, leptin and liver enzymes but lower adiponectin and HDL-C levels than the healthy controls. (p<0.05 for all, **Table 1**). No differences were found in physical activity, dietary energy or energy yield nutrient intakes between the NAFLD and healthy control groups.

**Table 1:** Physical characteristics, fat mass distribution, glucose metabolism hormones and liver enzymes in the healthy controls and NAFLD group (MIXED model estimated marginal means with 95% confidence intervals are given taking into account shared environment within family (husband and wife) and contrast estimates’ p-values were used to localize the significant differences between the two groups and group by gender interaction).

	****Healthy controls (n = 71)****		****NAFLD (n = 30)****			
	****Mean****	95% CI	Mean	95% CI	p	Group by gender
**Men/women (n)**	31/40		18/12		0.372	
**Age (years)**	51.7	(50.5, 52.9)	52.9	(50.9, 54.8)	0.244	0.479
**Height (cm)**	171.4	(170.1, 172.8)	171.6	(169.5, 173.8)	0.480	0.382
**Weight (kg)**	73.2	(70.7, 75.7)	87.3	(83.3, 91.3)	<0.001	0.493
**BMI**	24.9	(24.0, 25.7)	29.6	(28.3, 30.9)	<0.001	0.516
**FM (kg)**	19.8	(18.0, 21.6)	30.8	(27.9, 33.8)	<0.001	0.209
**SAT (kg)**	2.82	(2.49, 3.15)	4.61	(4.08, 5.15)	<0.001	0.002
**VAT (kg)**	0.749	(0.679, 0.818)	1.1	(0.99, 1.21)	0.005	0.293
**RAT (kg)**	1.13	(0.978, 1.28)	2.2	(2.00, 2.44)	<0.001	0.470
**IMCL (%)**	0.16	(0.142, 0.179)	0.24	(0.212, 0.268)	0.001	0.780
**EMCL (%)**	0.345	(0.281, 0.409)	0.408	(0.300, 0.515)	0.139	0.216
**Energy (kcal/day)**	1979	(1963, 2095)	1958	(1759, 2157)	0.674	0.947
**Protein (E%)**	18.1	(17.4, 18.8)	18.8	(17.5, 20.0)	0.557	0.980
**Fat_tot_ (E%)**	33.5	(31.8, 35.2)	31.7	(28.7, 34.5)	0.256	0.509
**SAFA (E%)**	12.9	(12.1, 13.7)	12.5	(11.1, 13.8)	0.588	0.766
**MUFA (E%)**	11.4	(10.6, 12.3)	10.4	(8.96, 11.9)	0.300	0.618
**PUFA (E%)**	6.00	(5.53, 6.45)	5.43	(4.63, 6.23)	0.100	0.170
**Ch (E%)**	45.8	(43.9, 47.7)	47.2	(43.9, 50.5)	0.359	0.484
**Sucrose (E%)**	5.02	(4.09, 5.95)	6.72	(5.12, 8.31)	0.515	0.419
**PA (time/week)**	2.94	(2.53, 3.35)	2.2	(1.55, 2.86)	0.143	0.806
**PA (hour/week)**	3.81	(3.36, 4.26)	3.25	(2.54, 3.97)	0.183	0.525
**Glucose (mmol/l)**	5.46	(5.33, 5.59)	5.71	(5.49, 5.92)	0.030	0.239
**Insulin (μIU/ml)**	6.22	(5.12, 7.32)	10.3	(8.58, 12.1)	0.001	0.306
**HOMA-IR**	1.53	(1.24, 1.82)	2.68	(2.21, 3.15)	<0.001	0.215
**hsCRP (ng/ml)**	722	(376, 1068)	1562	(1020, 2103)	0.006	0.709
**NEFA (μmol/l)**	395	(353, 438)	453	(383, 524)	0.032	0.069
**Serum-TG**	1.03	(0.93, 1.12)	1.45	(1.30, 1.59)	<0.001	0.067
**Leptin (ng/ml)**	11.9	(7.8, 16.0)	30.9	(24.8, 37.0)	<0.001	0.001
**Adiponectin (μg/ml)**	9.4	(7.8, 11.0)	5.1	(2.6, 7.6)	0.011	0.103
**ALP (IU/l)**	60.5	(56.9, 64.1)	64.9	(58.9, 70.9)	0.299	0.771
**ALT (IU/l)**	18.9	(16.0, 21.8)	30.9	(26.3, 35.5)	<0.001	0.129
**AST (IU/l)**	20.1	(18.6, 21.7)	23.0	(20.6, 24.4)	0.003	0.020
**GGT (IU/l)**	29.6	(25.0, 34.2)	39.4	(32.0, 46.8)	0.009	0.103

NAFLD = non-alcohol fatty liver disease; BMI = body mass index; FM = fat mass of the whole body; SAT = abdominal subcutaneous adipose tissue; VAT = visceral adipose tissue; RAT = retroperitoneal adipose tissue; IMCL = intra-myocellular lipids; EMCL = extra-myocellular lipids; E = energy; SAFA = saturated fatty acids; MUFA = monounsaturated fatty acids; PUFA = polyunsaturated fatty acids; Ch = carbohydrate; PA = physical activity; hsCRP = high-sensitivity C-reactive protein; NEFA = non-esterified fatty acids; TG = triglycerides; ALP = alkaline phosphatase; ALT = alanine aminotransferase; AST = aspartate aminotransferase; GGT = γ-glutamyltransferase.

### Serum metabolites

A cluster analysis of serum metabolites is illustrated in **S1 Fig.** The analysis revealed increased levels of very-low density lipoprotein (VLDL) subclasses, mono-unsaturated fatty acids, gluconeogenic substrates, orosomucoid and branched-chain amino acids, and decreased levels of high-density lipoprotein subclasses in participants with NAFLD. All essayed metabolites and lipoprotein subclass quantities and statistics are shown in **S1 Table**.

To further identify relevant metabolites associated with NAFLD, we used principal component analysis. Mean metabolite component levels are shown in **Table 2**. Factor 1 (omega 7and 9 and saturated fatty acids, total fatty acids and mono-unsaturated fatty acids), factor 2 (isoleucine, leucine, valine, phenylalanine, tyrosine and orosomucoid) and factor 3 (acetate, alanine, lactate, pyruvate) were significantly higher in the NAFLD group compared to the healthy control group (p = 0.008 to p<0.001). No group by gender interaction was found in any of the factors.

**Table 2:** Mean metabolite component levels stratified by the healthy control and NAFLD groups (MIXED model estimated marginal means with 95% confidence intervals are given taking into account shared environment within family, and contrast estimates’ p-values were used to localize the significant differences between the two groups and group by gender interaction).

	****Healthy Controls (n = 71)****		****NAFLD (n = 30)****			
	Mean	95% CI	Mean	95% CI	p	Group by Gender
**Factor 1**	-0.233	(-0.471, 0.006)	0.640	(0.268, 1.011)	<0.001	0.171
**Factor 2**	-0.235	(-0.450, -0.019)	0.610	(0.277, 0.943)	0.001	0.450
**Factor 3**	-0.216	(-0.463, 0.031)	0.492	(0.111, 0.874)	0.008	0.368
**Factor 4**	-0.049	(-0.303, 0.204)	-0.051	(-0.446, 0.343)	0.996	0.988
**Factor 5**	0.015	(-0.260, 0.290)	-0.054	(-0.479, 0.371)	0.938	0.879
**Factor 6**	0.093	(-0.179, 0.365)	-0.173	(-0.593, 0.248)	0.753	0.558

NAFLD = non-alcohol fatty liver disease; values are given as mean and 95% confident interval (CI). Factor 1 (Omega 7 and 9 and saturated fatty acids, total fatty acids, mono-unsaturated fatty acids); Factor 2 (isoleucine, leucine, valine, phenylalanine, tyrosine and orosomucoid); Factor 3 (acetate, alanine, lactate, pyruvate); Factor 4 (esterified cholesterol, free cholesterol, omega 6 fatty acids, phosphoglycerides, phosphocholines and sphingomyelines); Factor 5 (beta-hydroxybutyrate, citrate, histidine); Factor 6 (acetoacetate, glutamine)

The mean metabolite component levels were further compared between the quintiles of liver fat content adjusting for gender, BMI, visceral fat mass, leptin and adiponectin (**S2 Table**). Factor 1 was significantly higher in the 5^th^ quintile compared to the 1^st^ and 2^nd^ quintiles. Factor 2 was significantly higher in the 3^rd^, 4^th^ and 5^th^ quintile compared to the 1^st^ quintile, and factor 4 was significantly higher in the 5^th^ quintile compared to 1^st^ quintile. No significant difference in the other factors was observed between the highest and lowest quintile groups.

### Adipose tissue gene expression

To elucidate the metabolic pathways associated with NAFLD, we studied global transcript profiles of adipose tissue and skeletal muscle. Microarray analysis revealed 709 differential expressed genes (adjusted p<0.05) in the adipose tissue of the NAFLD group. Of these 709 genes, 255 were up-regulated and 454 were down-regulated (**S3 Table**). Kyoto Encyclopedia of Genes and Genomes (KEGG) enrichment analysis of the differentially expressed genes (p < 0.05) identified 6 down regulated pathways (**Table 3)**. The most down-regulated pathway was valine, leucine and isoleucine degradation (p = 4.6x10^-9^). Down-regulated genes in this pathway included the mitochondrial components (BCKDHB and DLD) that are common to the degradation of all BCAAs, i.e., isoleucine, leucine and valine. Genes specific for the degradation of leucine (AUH), isoleucine (PCCA and PCCB) and valine (HIBADH) were also down-regulated in the subjects with NAFLD.

**Table 3:** KEGG pathway enrichment analysis of differentially expressed genes in adipose tissue.

P-value	Count	Size	Pathway name	Genes
4.6x10-9	18	44	Valine, leucine and isoleucine degradation	ACADM, ACADSB, ALDH7A1, ALDH9A1, AUH, BCKDHB, DLD, HADH, HADHA, HADHB, HIBADH, HIBCH, MCCC1, MCEE, MUT, OXCT1, PCCA, PCCB
3.0x10-7	13	30	Citrate cycle (TCA cycle)	CS, DLD, DLST, FH, IDH1, IDH3A, IDH3B, PCK1, PDHB, SDHB, SUCLA2, SUCLG1, SUCLG2
3.3x10-5	13	43	Fatty acid degradation	ACADM, ACADSB, ACADVL, ACSL1, ADH1B, ADH5, ALDH7A1, ALDH9A1, DCI, HADH, HADHA, HADHB, PECI
7.1x10-3	20	132	Oxidative phosphorylation	ATP5A1, ATP5B, ATP5G3, ATP5L, ATP6AP1, ATP6V1C1, COX5A, COX5B, CYC1, NDUFA10, NDUFA12, NDUFA6, NDUFB4, NDUFB5, NDUFB6, NDUFS1, NDUFS2, NDUFS4, SDHB, UQCRC2
1.9x10-2	11	65	Glycolysis / Gluconeogenesis	ADH1B, ADH5, ALDH7A1, ALDH9A1, DLD, ENO1, PCK1, PDHB, PFKP, PGK1, PGM1
3.5x10-2	12	80	Glycerophospholipid metabolism	AGPAT6, AGPAT9, CEPT1, CHKA, CHPT1, CRLS1, ETNK2, GNPAT, GPD1L, GPD2, LPCAT1, PGS1

Count = Amount of differentially expressed genes that mapped on pathway. Size = Total amount of genes involved in pathway.

**Fig 1:**
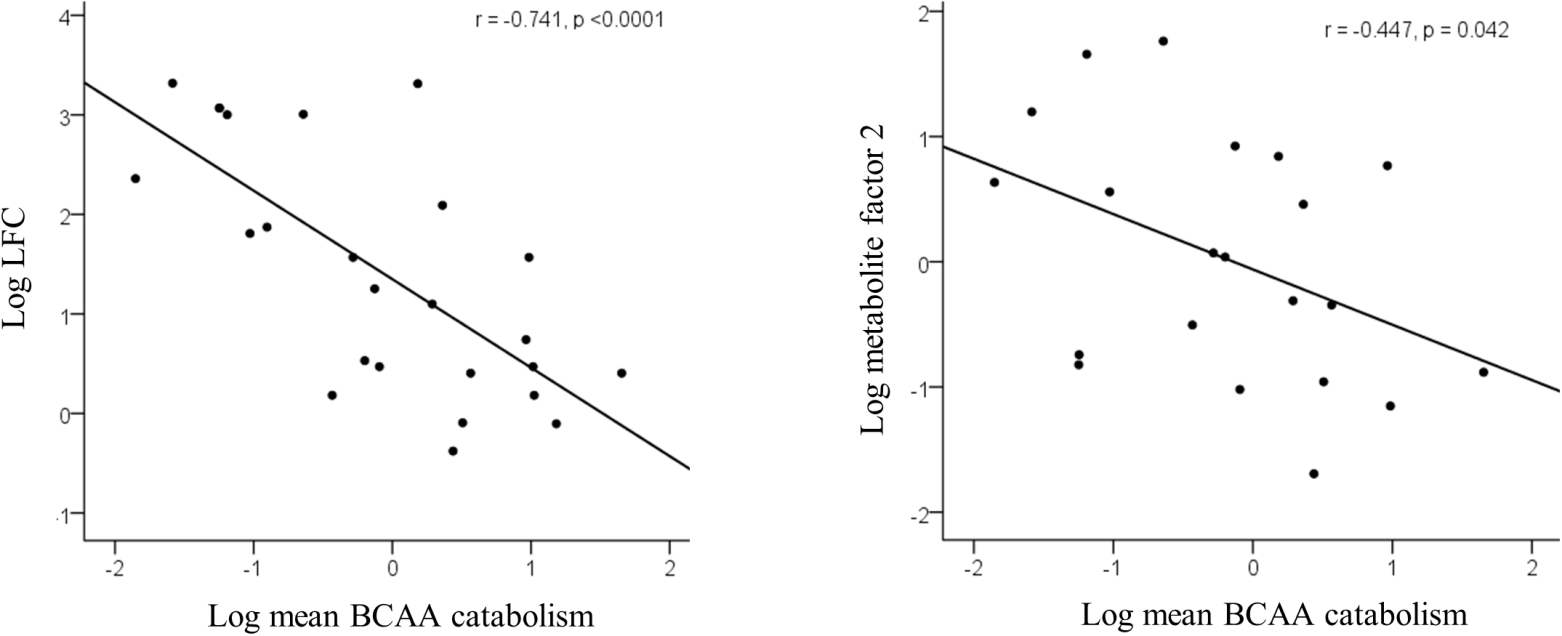
Correlations between liver fat content (LFC) assessed by ^1^H MRS and different adipose tissue gene expression clusters in certain pathways. The LFC was transformed into a normal distribution by natural logarithms. Each dot represents an individual and the line is a linear regression fit line.

**Fig 2:**
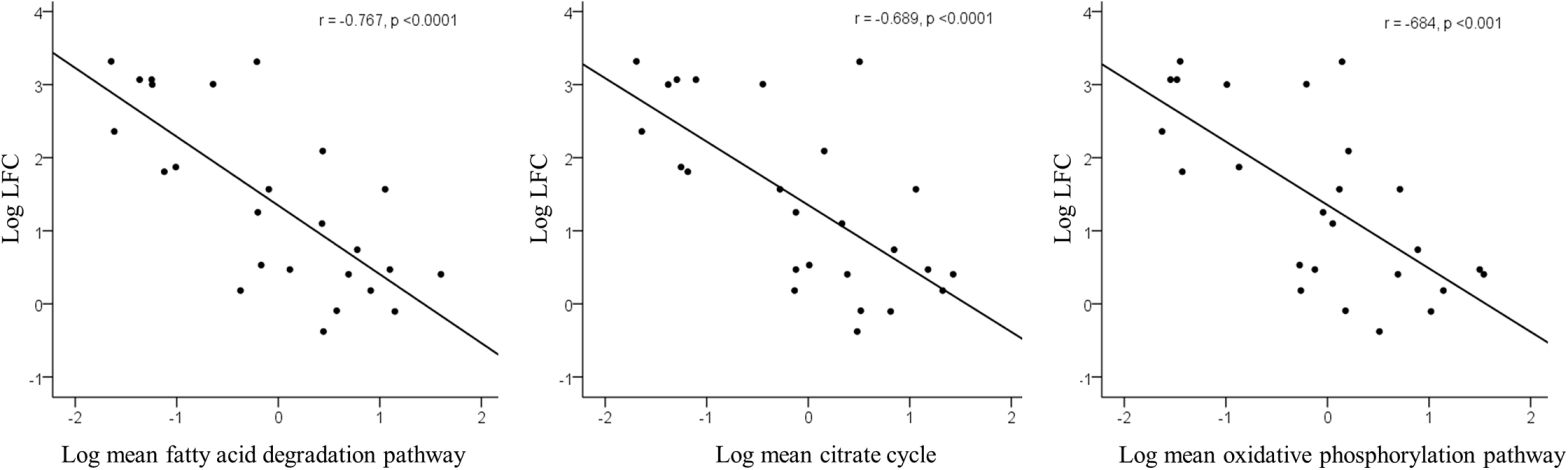
Correlations between serum metabolites factor 2 (isoleucine, leucine, valine, phenylalanine, tyrosine and orosomucoid) and different adipose tissue gene expression clusters in certain pathways. Each dot represents an individual and the line is a linear regression fit line.

The mean centroid of the BCAA degradation pathway correlated negatively with liver fat content and serum metabolite factor 2 (Fig 1), serum total BCAA (r = -0.471, p = 0.023) and fasting insulin concentrations (r = -0.550, p = 0.008) and HOMA-IR (r = -0.542, p = 0.009). In a multiple linear regression analysis, including total FM, visceral FM, retroperitoneal FM, HOMA-IR and hsCRP, only the BCAA degradation pathway and serum metabolite factor 2 remained significantly associated with liver fat (β = -0.791, and β = 0.992, respectively, p<0.05 for both). Non-alcoholic fatty liver was also associated with significant down-regulation of the energy metabolism in the adipose tissue. Fatty acid degradation, citric acid cycle and oxidative phosphorylation were associated with liver fat (r values ranged from -0.684 to -0.767, p<0.001 for all, **Fig 2**). No significant associations were found between the gene pathways and other serum metabolite factors (data not shown).

### Skeletal muscle gene expression and signaling protein phosphorylation

Unexpectedly, no differentially expressed genes were found in the skeletal muscle. However, since skeletal muscle is the primary site of insulin-stimulated glucose disposal, we further studied whether there were differences in the phosphorylation levels of several signaling proteins related to glucose metabolism. No differences in the phosphorylation levels of insulin receptor β and its downstream targets of Akt, ERK1/2, or mTOR and 4EBP1 were found. The level of phosphorylated AS160, which promotes translocation of glucose transporters to the cell membrane, was also similar between the groups.

## Discussion

In this study, we showed that increased serum branched-chain and aromatic amino acids are already present well-below the clinical cutoff value for NAFLD. In addition, reduced BCAA catabolism and mitochondrial energy metabolism were observed in the adipose tissue of the NAFLD group, whereas no significant between-group differences in skeletal muscle gene expression were found.

The pathophysiology of NAFLD is complex and has not been fully elucidated. However, there is growing evidence that obesity is an important factor in its causation [37]. Recent studies suggest that the major basis for this link is the ability of obesity to engender insulin resistance [12]. The current theory suggests that as the adipose tissue mass expands, chronic inflammation in the adipose tissue ensues [38]. The inflamed adipose tissue becomes insulin resistant, and the impaired ability of insulin to suppress lipolysis leads to increased flux of non-esterified fatty acids, which accumulate in the liver as triglycerides [39]. The excessive accumulation of intrahepatic triglycerides gradually attenuates the ability of insulin to suppress hepatic gluconeogenesis and triglyceride synthesis, resulting in the development of hyperglycemia, hyperinsulinemia and dyslipidemia [40]. However, studies have suggested that NAFLD can also develop in the absence of marked insulin resistance and increased adipose tissue lipolysis [41], and that hepatokines may be involved in the cross-talk between liver and extra-hepatic tissues [42]. The results of the present study showed that all the measures of adiposity, serum glucose, insulin and HOMA-IR as well as free fatty acids, triglycerides and CRP were higher in the NAFLD group compared to healthy controls. Elevated concentrations of gluconeogenetic substrates, VLDL triglycerides and various species of fatty acids further supported the notion of increased gluconeogenetic activity and imbalanced lipid metabolism in the subjects with NAFLD.

Significant increases in serum BCAA and aromatic amino acid concentrations were found in the subjects with NAFLD compared to those with low liver fat content. These findings demonstrate the perturbations in systemic amino acid homeostasis that accompany liver fat accretion. Recent studies have also shown increased serum BCAA in subjects with NAFLD [7,9,43]. However, these studies were conducted in morbidly obese patients undergoing bariatric surgery [9], or in subjects with type 2 diabetes using non-specific ultrasonography [7], which is substantially limited by its low sensitivity to mild steatosis and inability to provide reliable quantitative information on liver fat infiltration [43]. Another study found increased BCAA in the plasma of subjects with NASH but not NAFLD when compared to healthy controls [44]. However, no significant difference in BCAA was found between NAFLD and NASH, which is not surprising given that steatosis and steatohepatitis are defects on a continuum. Furthermore, although biopsy is considered the “golden standard” in comparative studies of fatty liver disease, sampling errors have been shown to limit its diagnostic accuracy [45]. Thus, one should bear in mind the possibility of methodological errors when interpreting dichotomized data, especially when the variable under investigation is a continuous variable such as liver fat [46]. Here, we used ^1^H magnetic resonance spectroscopy, which provides a highly specific estimation of hepatic fat *in vivo* [30]. To overcome the possibility of misclassification, we also divided our participants into quintile groups based on liver fat content. The results showed that the level of the metabolite factor consisting of BCAA and aromatic amino acids was significantly elevated at mean liver fat content of 2.4% (**S2 Table**), which is well below the clinical diagnostic cut-off value. This finding suggests that clinically meaningful hepatic steatosis could be present even at less than 5% liver fat content. Importantly, we have previously demonstrated with another study cohort that the same metabolites as in metabolite factor 2 (BCAA and tyrosine and phenylalanine) were auspicious biomarkers determining metabolic health independent of obesity and physical activity [34]. Other studies have also reported associations between systemic BCAA and metabolic health [47–49]. Although we cannot fully explain the discrepancy between our study and the other studies in te the state and progression of NAFLD, our results suggest that perturbations in systemic BCAA homeostasis could be an early event in the development of NAFLD. This notion is supported by a recent study which demonstrated that chronic elevation of circulating BCAA induces hepatic mitochondrial dysfunction in NAFLD [50].

We further searched for signs of early changes in metabolic pathways in the adipose tissue and skeletal muscle. We found a significant reduction in the adipose tissue BCAA catabolism pathway in subjects with NAFLD compared to those with low liver fat content. The decrease in BCAA catabolism was inversely associated with the serum total BCAA, serum metabolite factor 2, fasting insulin, HOMA-IR and liver fat content. These findings are in line with an earlier study in monozygotic twins discordant for obesity, which showed that down-regulation of BCAA catabolism in subcutaneous adipose tissue was associated with increased insulin resistance and liver fat content [22]. In our study, liver fat accumulation was also associated with significant down-regulation of the energy metabolism in the adipose tissue. Thus it is possible that decreased BCAA catabolism and impaired mitochondrial function in subcutaneous adipose tissue could link excess adiposity to the development of insulin resistance and liver fat accumulation [51].

The down-regulation of BCAA catabolism in the present study could be ascribed to reduced mitochondrial respiration, as indicated by the concurrent significant down-regulation of the TCA cycle, oxidative phosphorylation and decreased fatty acid degradation in the adipose tissue. These metabolic impairments could also be attributable to local inflammation induced by excessive enlargement of adipocytes or diminished adipocyte differentiation [52]. A study with monozygotic twins showed marked inflammation in the subcutaneous adipose tissue concurrently with decreased BCAA catabolism in obese subjects [22]. Inflammation-induced regulation of BCAA metabolism in visceral, but not subcutaneous adipose tissue was also recently reported [53]. In the present study, inflammatory pathways were not significantly up-regulated in the adipose tissue of the participants with NAFLD. However, the two most over-expressed genes in the adipose tissue were chitinase-3-like protein 1 (CHI3L1) and matrix metallopeptidase 9 (MMP9) (**S3 Table**). These genes are related to cytoskeleton re-organization and degradation of the extracellular matrix and have been suggested to cause inflammatory cell infiltration, resulting in persistent inflammation in the adipose tissue [54]. These results are consistent with the higher serum hsCRP and orosomucoid observed in subjects with NAFLD, indicating the presence of subclinical low grade inflammation.

In addition to adipose tissue dysfunction, recent studies have implicated increased intramyocellular lipid content [55], increased muscle insulin resistance [16] and impaired skeletal muscle energy metabolism [56] in hepatic steatosis. In the present study, significantly higher intramuscular lipid content was observed in the NAFLD group compared to the low liver fat content group. Unexpectedly, no difference between the groups in gene expression profiles in the skeletal muscle was found. To confirm these findings with respect to glucose metabolism, we further studied whether there were differences in the phosphorylation levels of several signaling proteins. No significant differences in the phosphorylation levels of insulin receptor β and its downstream targets of Akt, ERK1/2, or mTOR and 4EBP1 were found. Nor was there any difference in the level of phosphorylated AS160, which promotes translocation of glucose transporters to the cell membrane. These findings by no means negate the role of skeletal muscle insulin resistance in the development of systemic metabolic disorders. However, these findings do suggest that, at least in the fasting state, skeletal muscle glucose metabolism is not altered in the early stages of NAFLD, irrespective of increased intra-myocellular lipid content.

Our study is not without limitations. We acknowledge that our approach cannot determine causality and that the results can indicate only a general pattern. The study was conducted during 2009 to 2010. The interval between the different data collection points for each study participant varied to some extent, ranging from a few days to several months. However, there was no difference between the healthy control and NAFLD groups in the sampling time window. Further, no change in body weight or body composition was observed during the study period, nor was there any change in diet. The study participants were married couples drawn from a comprehensive and carefully performed family study. The couples shared the same family environment and living conditions. They were carefully selected in order to minimize confounding factors. For these reasons, we are confident that our results are not biased by background characteristics and health history. However, the narrow age range of the subjects in our study (50–55 years) may partly explain the lack of agreement between some of our findings and data from previous studies, and thus suggest that our results should be interpreted in the context of age. It should also be noted that this study is limited by the fact that adipose tissue and skeletal muscle biopsies were only available for a relatively small group of subjects (11 with and 21 without NAFLD). However, we used the state-of-the-art method ^1^H MRS to quantify the ectopic fat content and serum metabolites. Furthermore, all the NAFLD subjects were in early or in moderate stage of fatty liver, which gave us the possibility to identify biomarkers associated with NAFLD in its early stage.

In summary, we demonstrate that already in its early stage liver fat accumulation is associated with increased serum branched-chain and aromatic amino acids. Significant down-regulation of BCAA catabolism and mitochondrial energy metabolism in the adipose tissue was found in participants with NAFLD, whereas no aberrant gene expression in skeletal muscle was observed. The observed associations between decreased BCAA catabolic activity and serum metabolite factor 2, total BCAA concentration and liver fat content suggest that adipose tissue dysfunction may play a key role in the systemic nature of NAFLD pathogenesis. However, whether BCAAs are involved in the development of NAFLD in a functional manner is unclear and warrants further study.

## Supporting Information

S1 Fig The heat map shows changes in the x-fold standard deviation from the overall mean concentration of the metabolite in each individual from either the non-alcohol fatty liver (NAFLD) or healthy control group. Green squares represent a decrease and red squares an increase in values. Metabolite names are shown on the x-axis and individual subjects with adherent groups on the (right) y-axis. The metabolite names are shown in S4 Table.
(PPTX)

S1 Table Metabolites between the healthy control and NAFLD groups in men and women. (DOCX)

S2 Table Mean metabolite component levels stratified by the quintiles of liver fat content (General linear model estimated marginal means with 95% confidence intervals adjusted for BMI, visceral fat mass, leptin and adiponectin). (DOCX)

S3 Table Differentially expressed genes in the adipose tissue of the NAFL group. (DOCX)

S4 Table Abbreviations and full names of metabolites. (DOCX)

## Abbreviations

NAFLD: non-alcoholic fatty liver disease
^1^H MRS: = proton magnetic resonance
NMR: nuclear magnetic resonance
BCAA: branched-chain amino acids
AAA: aromatic amino acids
NASH: non-alcoholic steatohepatitis
LFC: liver fat content
BMI: body mass index
PA: participation in leisure time physical activity
FM: fat mass
SAT: abdominal subcutaneous adipose tissue
VAT: visceral adipose tissue
RAT: retroperitoneal adipose tissue
IMCL: Muscle intra-myocellular lipid
EMCL: extra-myocellular lipid
ALP: alkaline phosphatase
ALT: alanine aminotransferase
AST: aspartate aminotransferase
GGT: γ-glutamyl transferase
NEFA: non-esterified fatty acids
HOMA-IR index: homeostatic model assessment of insulin resistance
TG: triglycerides
VLDL: very low density lipoprotein
E: energy
SAFA: saturated fatty acids
MUFA: monounsaturated fatty acids
PUFA: polyunsaturated fatty acids
Ch: carbohydrate
FM: fat mass
hsCRP: high-sensitivity C-reactive protein
Factor 1: Omega 7and 9 and saturated fatty acids, total fatty acids, mono-unsaturated fatty acids
Factor 2: isoleucine, leucine, valine, phenylalanine, tyrosine and orosomucoid
Factor 3: acetate, alanine, lactate, pyruvate
Factor 4: esterified cholesterol, free cholesterol, omega 6 fatty acids, phosphoglycerides, phosphocholines and sphingomyelines
Factor 5: beta-hydroxybutyrate, citrate, histidine
Factor 6: acetoacetate, glutamine
KEGG: Kyoto Encyclopedia of Genes and Genomes
