# Supplementary material for: Adipose Tissue Dysfunction and Altered Systemic Amino Acid Metabolism Are Associated with Non-Alcoholic Fatty Liver Disease
Source: PLoS One. 2015 Oct 6;10(10):e0138889. doi: 10.1371/journal.pone.0138889 (PMC4595021; doi:10.1371/journal.pone.0138889)
Supplement: S1 Fig — Green squares represent a decrease and red squares an increase in values. Metabolite names are shown on the x-axis and individual subjects with adherent groups on the (right) y-axis. The metabolite names are shown in S4 Table. (PPTX) [file pone.0138889.s001.pptx]

## Slide 1
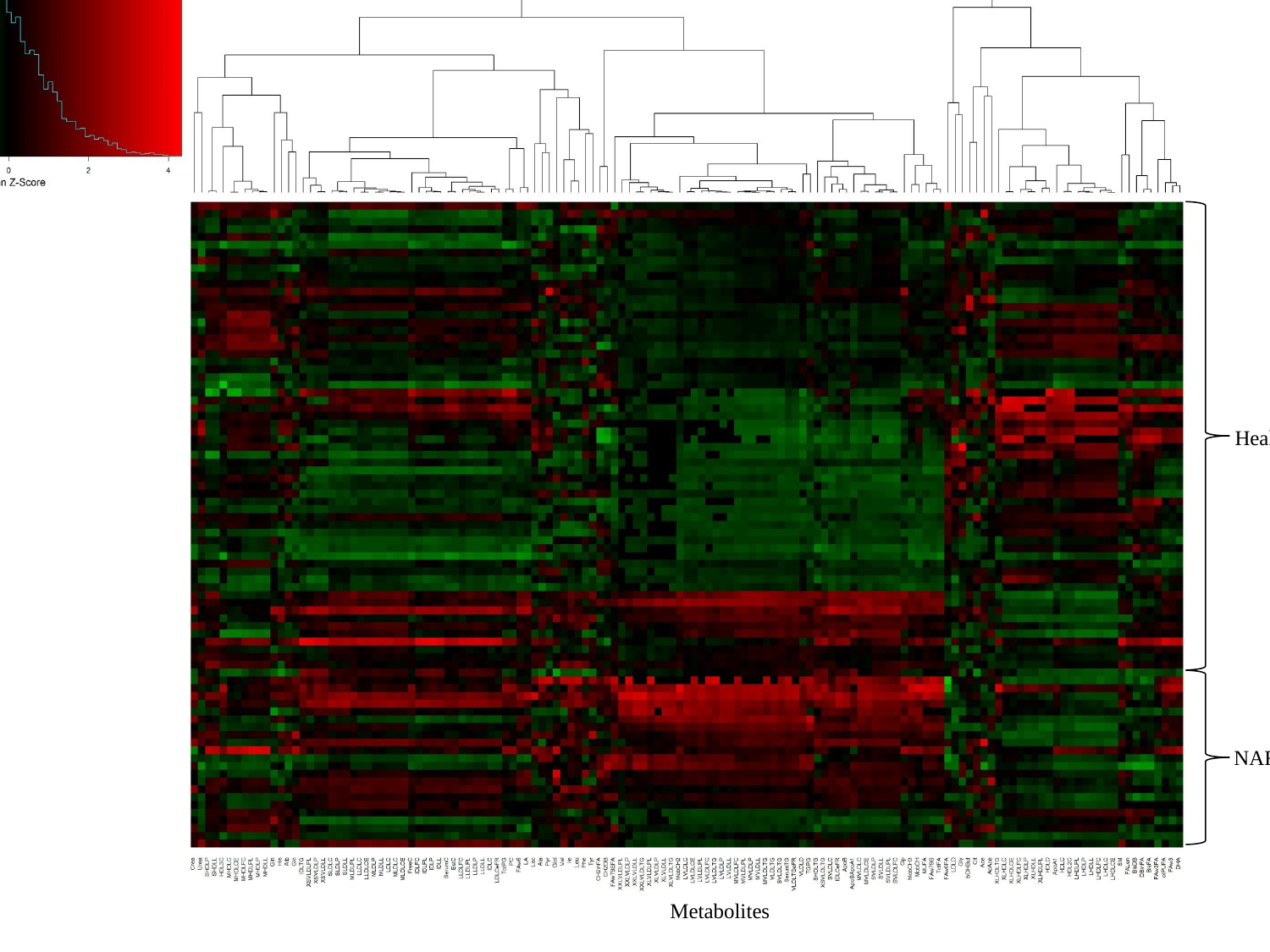

Figure 1 Hierarchical clustering of metabolomics data values in NAFLD and healthy control groups.
Healthy controls
NAFLD
Metabolites
