# Supplementary material for: Adipose Tissue Dysfunction and Altered Systemic Amino Acid Metabolism Are Associated with Non-Alcoholic Fatty Liver Disease
Source: PLoS One. 2015 Oct 6;10(10):e0138889. doi: 10.1371/journal.pone.0138889 (PMC4595021; doi:10.1371/journal.pone.0138889)
Supplement: S1 Table — (DOCX) [file pone.0138889.s002.docx]

**Supplementary Table 1**. Metabolites between the healthy control and NAFLD groups in men and women

|  | Healthy controls  (n=71) | | | NAFLD  (n=30) | | | | | |  | | |  | | | | |
| --- | --- | --- | --- | --- | --- | --- | --- | --- | --- | --- | --- | --- | --- | --- | --- | --- | --- |
| **Metabolites** | Mean | | SD | Mean | | | SD | | | p BH | | | Group by gender | | | | |
| **Lipoprotein subclasses** |  | |  |  | | |  | |  | | |  | | | | |  |
| albumine | 0.084 | 0.007 | | 0.085 | | 0.007 | | | | 0.663 | | | 0.957 | |  |  |  |
| XXL-VLDL-P | 0.061 | 0.061 | | 0.146 | | 0.107 | | | | **0.003** | | | 0.388 | |  |  |  |
| XXL-VLDL-L | 0.012 | 0.011 | | 0.025 | | 0.021 | | | | **0.014** | | | 0.165 | |  |  |  |
| XXL-VLDL-PL | 0.002 | 0.001 | | 0.003 | | 0.002 | | | | **0.023** | | | 0.359 | |  |  |  |
| XXL-VLDL-TG | 0.008 | 0.008 | | 0.018 | | 0.015 | | | | **0.008** | | | 0.153 | |  |  |  |
| XL-VLDL-P | 0.330 | 0.334 | | 0.713 | | 0.484 | | | | **0.007** | | | 0.204 | |  |  |  |
| XL-VLDL-L | 0.036 | 0.033 | | 0.072 | | 0.053 | | | | **0.010** | | | 0.392 | |  |  |  |
| XL-VLDL-PL | 0.006 | 0.006 | | 0.014 | | 0.011 | | | | **0.007** | | | 0.609 | |  |  |  |
| XL-VLDL-TG | 0.022 | 0.021 | | 0.045 | | 0.034 | | | | **0.010** | | | 0.396 | |  |  |  |
| L-VLDL-P | 2.358 | 2.125 | | 4.818 | | 2.840 | | | | **0.002** | | | 0.075 | |  |  |  |
| L-VLDL-L | 0.150 | 0.132 | | 0.299 | | 0.177 | | | | **0.002** | | | 0.089 | |  |  |  |
| L-VLDL-PL | 0.026 | 0.025 | | 0.055 | | 0.033 | | | | **0.002** | | | 0.126 | |  |  |  |
| L-VLDL-C | 0.035 | 0.029 | | 0.068 | | 0.040 | | | | **0.003** | | | **0.007** | |  |  |  |
| L-VLDL-CE | 0.020 | 0.015 | | 0.036 | | 0.021 | | | | **0.003** | | | 0.075 | |  |  |  |
| L-VLDL-FC | 0.016 | 0.014 | | 0.034 | | 0.023 | | | | **0.003** | | | **0.044** | |  |  |  |
| L-VLDL-TG | 0.090 | 0.076 | | 0.185 | | 0.118 | | | | **0.002** | | | **0.034** | |  |  |  |
| M-VLDL- P | 10.21 | 6.290 | | 16.35 | | 7.130 | | | | **0.001** | | | 0.051 | |  |  |  |
| M-VLDL-L | 0.375 | 0.218 | | 0.584 | | 0.248 | | | | **0.001** | | | 0.100 | |  |  |  |
| M-VLDL-PL | 0.077 | 0.043 | | 0.118 | | 0.047 | | | | **0.001** | | | 0.084 | |  |  |  |
| M-VLDL-C | 0.110 | 0.056 | | 0.168 | | 0.065 | | | | **0.001** | | | **0.046** | |  |  |  |
| M-VLDL-CE | 0.065 | 0.029 | | 0.093 | | 0.033 | | | | **0.001** | | | 0.088 | |  |  |  |
| M-VLDL-FC | 0.046 | 0.027 | | 0.075 | | 0.033 | | | | **0.001** | | | **0.020** | |  |  |  |
| M-VLDL-TG | 0.185 | 0.121 | | 0.314 | | 0.153 | | | | **0.001** | | | 0.052 | |  |  |  |
| S-VLDL-P | 22.62 | 7.984 | | 30.16 | | 8.186 | | | | **0.001** | | | **0.025** | |  |  |  |
| S-VLDL-L | 0.484 | 0.168 | | 0.641 | | 0.166 | | | | **0.001** | | | **0.023** | |  |  |  |
| S-VLDL-PL | 0.113 | 0.035 | | 0.145 | | 0.033 | | | | **0.001** | | | **0.023** | |  |  |  |
| S-VLDL-C | 0.178 | 0.058 | | 0.225 | | 0.053 | | | | **0.002** | | | **0.031** | |  |  |  |
| S-VLDL-FC | 0.071 | 0.024 | | 0.092 | | 0.022 | | | | **0.001** | | | **0.014** | |  |  |  |
| S-VLDL-TG | 0.192 | 0.079 | | 0.271 | | 0.087 | | | | **0.001** | | | **0.033** | |  |  |  |
| XS-VLDL-P | 0.031 | 0.007 | | 0.036 | | 0.007 | | | | **0.028** | | | 0.230 | |  |  |  |
| XS-VLDL-L | 0.450 | 0.105 | | 0.505 | | 0.096 | | | | **0.036** | | | 0.216 | |  |  |  |
| XS-VLDL-PL | 0.133 | 0.032 | | 0.144 | | 0.032 | | | | 0.182 | | | 0.443 | |  |  |  |
| XS-VLDL-TG | 0.105 | 0.028 | | 0.126 | | 0.027 | | | | **0.004** | | | 0.053 | |  |  |  |
| IDL-P | 0.079 | 0.017 | | 0.086 | | 0.017 | | | | 0.154 | | | 0.472 | |  |  |  |
| IDL-L | 0.968 | 0.205 | | 1.038 | | 0.205 | | | | 0.182 | | | 0.485 | |  |  |  |
| IDL-PL | 0.264 | 0.055 | | 0.282 | | 0.057 | | | | 0.205 | | | 0.656 | |  |  |  |
| IDL-C | 0.584 | 0.125 | | 0.616 | | 0.128 | | | | 0.322 | | | 0.534 | |  |  |  |
| IDL-FC | 0.182 | 0.039 | | 0.187 | | 0.043 | | | | 0.627 | | | 0.611 | |  |  |  |
| IDL-TG | 0.117 | 0.027 | | 0.128 | | 0.026 | | | | 0.107 | | | 0.186 | |  |  |  |
| L-LDL-P | 0.130 | 0.027 | | 0.141 | | 0.028 | | | | 0.130 | | | 0.368 | |  |  |  |
| L-LDL-L | 1.154 | 0.246 | | 1.251 | | 0.259 | | | | 0.135 | | | 0.379 | |  |  |  |
| L-LDL-PL | 0.286 | 0.053 | | 0.308 | | 0.053 | | | | 0.122 | | | 0.575 | |  |  |  |
| L-LDL-C | 0.758 | 0.173 | | 0.823 | | 0.187 | | | | 0.154 | | | 0.390 | |  |  |  |
| L-LDL-CE | 0.535 | 0.130 | | 0.589 | | 0.140 | | | | 0.117 | | | 0.347 | |  |  |  |
| L-LDL-FC | 0.224 | 0.043 | | 0.233 | | 0.047 | | | | 0.433 | | | 0.640 | |  |  |  |
| M-LDL-P | 0.106 | 0.023 | | 0.117 | | 0.024 | | | | 0.074 | | | 0.404 | |  |  |  |
| M-LDL-L | 0.676 | 0.154 | | 0.748 | | 0.164 | | | | 0.076 | | | 0.421 | |  |  |  |
| M-LDL-PL | 0.177 | 0.031 | | 0.194 | | 0.032 | | | | **0.039** | | | 0.522 | |  |  |  |
| M-LDL-C | 0.444 | 0.110 | | 0.494 | | 0.122 | | | | 0.092 | | | 0.456 | |  |  |  |
| M-LDL-CE | 0.314 | 0.087 | | 0.355 | | 0.097 | | | | 0.082 | | | 0.428 | |  |  |  |
| S-LDL-P | 0.127 | 0.027 | | 0.142 | | 0.029 | | | | **0.028** | | | 0.138 | |  |  |  |
| S-LDL-L | 0.446 | 0.104 | | 0.507 | | 0.112 | | | | **0.027** | | | 0.146 | |  |  |  |
| S-LDL-C | 0.282 | 0.069 | | 0.320 | | 0.080 | | | | **0.039** | | | 0.150 | |  |  |  |
| XL-HDL-P | 0.393 | 0.167 | | 0.286 | | 0.092 | | | | **0.001** | | | 0.886 | |  |  |  |
| XL-HDL-L | 0.481 | 0.192 | | 0.367 | | 0.108 | | | | **0.003** | | | 0.831 | |  |  |  |
| XL-HDL-PL | 0.234 | 0.107 | | 0.161 | | 0.061 | | | | **0.001** | | | 0.828 | |  |  |  |
| XL-HDL-C | 0.244 | 0.101 | | 0.194 | | 0.055 | | | | **0.009** | | | 0.722 | |  |  |  |
| XL-HDL-CE | 0.179 | 0.071 | | 0.143 | | 0.040 | | | | **0.009** | | | 0.414 | |  |  |  |
| XL-HDL-FC | 0.063 | 0.028 | | 0.046 | | 0.015 | | | | **0.002** | | | 0.990 | |  |  |  |
| XL-HDL-TG | 0.013 | 0.004 | | 0.013 | | 0.003 | | | | 0.565 | | | 0.280 | |  |  |  |
| L-HDL-P | 1.034 | 0.396 | | 0.780 | | 0.307 | | | | **0.010** | | | 0.988 | |  |  |  |
| L-HDL-L | 0.801 | 0.317 | | 0.592 | | 0.240 | | | | **0.008** | | | 0.987 | |  |  |  |
| L-HDL-PL | 0.389 | 0.137 | | 0.305 | | 0.114 | | | | **0.014** | | | 0.996 | |  |  |  |
| L-HDL-C | 0.386 | 0.173 | | 0.266 | | 0.124 | | | | **0.005** | | | 0.933 | |  |  |  |
| L-HDL-CE | 0.300 | 0.133 | | 0.211 | | 0.096 | | | | **0.007** | | | 0.933 | |  |  |  |
| L-HDL-FC | 0.086 | 0.040 | | 0.054 | | 0.029 | | | | **0.002** | | | 0.864 | |  |  |  |
| M-HDL-P | 1.594 | 0.288 | | 1.679 | | 0.308 | | | | 0.266 | | | 0.567 | |  |  |  |
| M-HDL-L | 0.851 | 0.160 | | 0.889 | | 0.166 | | | | 0.366 | | | 0.580 | |  |  |  |
| M-HDL-PL | 0.396 | 0.068 | | 0.413 | | 0.076 | | | | 0.334 | | | 0.612 | |  |  |  |
| M-HDL-C | 0.419 | 0.091 | | 0.430 | | 0.086 | | | | 0.626 | | | 0.577 | |  |  |  |
| M-HDL-CE | 0.339 | 0.073 | | 0.347 | | 0.068 | | | | 0.631 | | | 0.591 | |  |  |  |
| M-HDL-FC | 0.079 | 0.018 | | 0.080 | | 0.019 | | | | 0.663 | | | 0.586 | |  |  |  |
| S-HDL-P | 3.929 | 0.379 | | 4.160 | | 0.342 | | | | **0.016** | | | 0.676 | |  |  |  |
| S-HDL-L | 1.037 | 0.114 | | 1.101 | | 0.107 | | | | **0.027** | | | 0.801 | |  |  |  |
| S-HDL-TG | 0.040 | 0.012 | | 0.048 | | 0.012 | | | | **0.013** | | | 0.715 | |  |  |  |
| VLDL-TG | 0.568 | 0.309 | | 0.891 | | 0.371 | | | | **0.001** | | | 0.058 | |  |  |  |
| LDL-C | 1.483 | 0.350 | | 1.640 | | 0.385 | | | | 0.094 | | | 0.271 | |  |  |  |
| HDL-C | 1.509 | 0.339 | | 1.327 | | 0.244 | | | | **0.026** | | | 0.963 | |  |  |  |
| Serum-C | 4.094 | 0.717 | | 4.249 | | 0.671 | | | | 0.396 | | | 0.329 | |  |  |  |
| VLDL-D | 35.77 | 1.194 | | 36.99 | | 1.334 | | | | **0.001** | | | **0.040** | |  |  |  |
| LDL-D | 23.51 | 0.160 | | 23.45 | | 0.149 | | | | 0.158 | | | 0.161 | |  |  |  |
| HDL-D | 10.04 | 0.289 | | 9.839 | | 0.165 | | | | **0.001** | | | 0.812 | |  |  |  |
| VLDL-TG-eFR | 0.528 | 0.254 | | 0.804 | | 0.315 | | | | **0.001** | | | 0.072 | |  |  |  |
| IDL-C-eFR | 0.158 | 0.052 | | 0.208 | | 0.060 | | | | **0.001** | | | 0.098 | |  |  |  |
| LDL-C-eFR | 2.430 | 0.453 | | 2.526 | | 0.469 | | | | 0.413 | | | 0.554 | |  |  |  |
| HDL2-C | 1.006 | 0.320 | | 0.818 | | 0.214 | | | | **0.014** | | | 0.811 | |  |  |  |
| HDL3-C | 0.503 | 0.042 | | 0.509 | | 0.043 | | | | 0.582 | | | 0.205 | |  |  |  |
| Apolipoprotein A1^a^ | 1.552 | 0.245 | | 1.483 | | 0.187 | | | | 0.240 | | | 0.342 | |  |  |  |
| Apolipoprotein B^a^ | 0.752 | 0.152 | | 0.878 | | 0.162 | | | | **0.002** | | | 0.142 | |  |  |  |
| ApoB/ApoA1 | 0.493 | 0.117 | | 0.598 | | 0.118 | | | | **0.001** | | | 0.415 | |  |  |  |
| **Lipid extract constituents** |  | | |  |  | | |  | | |  | | |  | |  |  |
| Mobile lipids Ch2 | 18.89 | 6.810 | | 25.94 | | 8.751 | | | | **0.000** | | | 0.073 | |  |  |  |
| Mobile lipids Ch3 | 7.314 | 1.480 | | 8.589 | | 1.738 | | | | **0.000** | | | **0.038** | |  |  |  |
| Omega7and 9 fatty acids | 5.194 | 1.209 | | 6.349 | | 1.519 | | | | **0.001** | | | 0.106 | |  |  |  |
| MUFA | 2.339 | 0.662 | | 2.972 | | 0.773 | | | | **0.001** | | | 0.087 | |  |  |  |
| Omega7and9/ total fatty acid ratio | 60.08 | 3.150 | | 63.16 | | 4.780 | | | | **0.011** | | | 0.062 | |  |  |  |
| Triglycerides/phosphoglycerides | 1.189 | 0.453 | | 1.644 | | 0.654 | | | | **0.007** | | | 0.503 | |  |  |  |
| Esterified cholesterol | 2.953 | 0.517 | | 3.070 | | 0.499 | | | | 0.389 | | | 0.294 | |  |  |  |
| Free cholesterol | 1.140 | 0.212 | | 1.171 | | 0.197 | | | | 0.572 | | | 0.524 | |  |  |  |
| Omega3 fatty acids | 0.413 | 0.166 | | 0.444 | | 0.187 | | | | 0.493 | | | 0.294 | |  |  |  |
| Omega6 fatty acids | 2.994 | 0.444 | | 3.181 | | 0.503 | | | | 0.124 | | | 0.625 | |  |  |  |
| Total fatty acids | 8.61 | 1.69 | | 10.04 | | 1.91 | | | | **0.001** | | | 0.092 | |  |  |  |
| Total phosphoglycerides | 0.648 | 0.143 | | 0.692 | | 0.109 | | | | 0.205 | | | 0.297 | |  |  |  |
| Total phosphocholines | 1.583 | 0.300 | | 1.652 | | 0.235 | | | | 0.354 | | | 0.344 | |  |  |  |
| Sphingomyelines | 0.213 | 0.046 | | 0.215 | | 0.043 | | | | 0.785 | | | 0.828 | |  |  |  |
| Omega3/total fatty acid ratio | 4.734 | 1.417 | | 4.419 | | 1.599 | | | | 0.413 | | | 0.598 | |  |  |  |
| Omega6/total fatty acid ratio | 35.18 | 3.225 | | 32.42 | | 4.861 | | | | **0.023** | | | 0.054 | |  |  |  |
| CH2 in total fatty acids | 9.547 | 0.200 | | 9.629 | | 0.210 | | | | 0.124 | | | 0.575 | |  |  |  |
| Linoleic acid | 2.525 | 0.402 | | 2.626 | | 0.491 | | | | 0.377 | | | 0.472 | |  |  |  |
| Other polyunsaturated fatty acids | 1.797 | 0.577 | | 1.994 | | 0.526 | | | | 0.182 | | | 0.313 | |  |  |  |
| Docosahexaenoic acid | 1.286 | 0.090 | | 1.241 | | 0.109 | | | | 0.074 | | | 0.216 | |  |  |  |
| fatty acid length | 17.98 | 0.206 | | 17.92 | | 0.197 | | | | 0.334 | | | 0.638 | |  |  |  |
| **Low-molecular weight metabolites** |  |  | |  | |  | | | |  | | |  | |  |  |  |
| Betahydroxybutyrate | 0.052 | 0.049 | | 0.047 | | 0.035 | | | | 0.695 | | | 0.865 | |  |  |  |
| Acetate | 0.051 | 0.069 | | 0.041 | | 0.012 | | | | **0.045** | | | 0.333 | |  |  |  |
| Acetoacetate | 0.039 | 0.019 | | 0.040 | | 0.022 | | | | **0.003** | | | 0.453 | |  |  |  |
| Alanine | 0.379 | 0.052 | | 0.408 | | 0.063 | | | | **0.002** | | | 0.391 | |  |  |  |
| Citrate | 0.100 | 0.018 | | 0.105 | | 0.017 | | | | 0.276 | | | 0.740 | |  |  |  |
| Creatinine | 0.057 | 0.010 | | 0.059 | | 0.015 | | | | 0.614 | | | 0.119 | |  |  |  |
| Glutamine | 0.556 | 0.055 | | 0.536 | | 0.062 | | | | 0.182 | | | 0.715 | |  |  |  |
| Glycerol | 0.060 | 0.021 | | 0.082 | | 0.028 | | | | **0.001** | | | **0.039** | |  |  |  |
| Glycine | 0.278 | 0.062 | | 0.247 | | 0.042 | | | | **0.033** | | | 0.152 | |  |  |  |
| Orosomucoid | 1.219 | 0.158 | | 1.374 | | 0.174 | | | | **0.001** | | | 0.140 | |  |  |  |
| Histidine | 0.051 | 0.007 | | 0.053 | | 0.009 | | | | 0.238 | | | 0.885 | |  |  |  |
| Isoleucine | 0.042 | 0.010 | | 0.054 | | 0.011 | | | | **0.000** | | | 0.387 | |  |  |  |
| Leucine | 0.066 | 0.012 | | 0.080 | | 0.013 | | | | **0.001** | | | 0.291 | |  |  |  |
| Valine | 0.171 | 0.030 | | 0.196 | | 0.035 | | | | **0.003** | | | 0.785 | |  |  |  |
| Phenylalanine | 0.063 | 0.008 | | 0.068 | | 0.008 | | | | **0.016** | | | 0.414 | |  |  |  |
| Pyruvate | 0.068 | 0.017 | | 0.078 | | 0.017 | | | | **0.030** | | | 0.745 | |  |  |  |
| Tyrosine | 0.044 | 0.008 | | 0.051 | | 0.010 | | | | **0.003** | | | 0.172 | |  |  |  |
| Urea | 0.058 | 0.023 | | 0.054 | | 0.024 | | | | 0.500 | | | 0.610 | |  |  |  |
| Lactate | 0.906 | 0.225 | | 1.000 | | 0.299 | | | | 0.147 | | | 0.681 | |  |  |  |

Mean (SD) concentrations of metabolites assayed in the present study. Concentrations are in mmol/l unless stated otherwise. P-values are for 2-tailed t-tests comparing concentrations between healthy controls and NAFLD (non-alcohol fatty liver disease) groups adjusted for multiple comparisons using Benjamin-Hochberg correction. Abbreviations: VLDL: very-low-density lipoprotein; IDL: intermediate –density lipoprotein; LDL: low-density lipoprotein; HDL: high-density lipoprotein; XXL: extremely large; XL: very large; L: large; M: medium; S: small; XS: very small; L: total protein lipids; PL: phospholipids; C: cholesterol; CE: cholesterol esters; FC: free cholesterol; TG: triglycerides; D: mean diameter; eFR=values estimated with the extended friedewald method; Bis/DB: bisallylic groups per double bond; ^a^=g/l. Significant differences are marked in bold.
