# Supplementary material for: Adipose Tissue Dysfunction and Altered Systemic Amino Acid Metabolism Are Associated with Non-Alcoholic Fatty Liver Disease
Source: PLoS One. 2015 Oct 6;10(10):e0138889. doi: 10.1371/journal.pone.0138889 (PMC4595021; doi:10.1371/journal.pone.0138889)
Supplement: S2 Table — (DOCX) [file pone.0138889.s003.docx]

**Supplementary Table 2.** Mean metabolite component levels stratified by the quintiles of liver fat content (General linear model estimated marginal means with 95% confidence intervals adjusted for BMI, visceral fat mass, leptin and adiponectin)

| Variable | Q1  n=19  Mean | 95% CI | Q2  n= 22  Mean | 95% CI | Q3  n=23  Mean | 95% CI | Q4  n=19  Mean | 95% CI | Q5  n=15  Mean | 95% CI |
| --- | --- | --- | --- | --- | --- | --- | --- | --- | --- | --- |
| Liver fat% | 0.732 | (-0.438, 1.902) | 1.347 | (0.205, 2.489) ^a^ | 2.356 | (1.122, 3.589) ^a,b^ | 5.611 | (4.470, 6.753) ^a,b,c^ | 17.326 | (16.092, 18.559) ^a,b,c,d^ |
| FAC1 | -0.304 | (-0.755, 0.147) | -0.347 | (-0.840, 0.146) | 0.084 | (-0.359, 0.527) | 0.096 | (-0.364, 0.555) | 0.485 | (-0.005, 0.976) ^a,b^ |
| FAC2 | -0.500 | (-0.929, -0.071) | -0.247 | (-0.697, 0.203) | 0.106 | (-0.304, 0.516)^a^ | 0.294 | (-0.144, 0.732) ^a,b^ | 0.348 | (-0.094, 0.790) ^a,b^ |
| FAC3 | 0.027 | (-0.512, 0.565) | -0.112 | (-0.667, 0.452) | -0.282 | (-0.796, 0.233) | -0.089 | (-0.638, 0.461) | 0.403 | (-0.152, 0.957) |
| FAC4 | -0.542 | (-1.052, -0.033) | -0.462 | (-1.018, 0.094) | 0.026 | (-0.029, 0.746) | 0.228 | (-0.029, 0.746) | 0.357 | (-0.197, 0.910)^a^ |
| FAC5 | -0.003 | (-0.583, 0.576) | 0.189 | (-0.418, 0.796) | -0.081 | (-0.634, 0.473) | 0.102 | (-0.490, 0.693) | -0.194 | (-0.790, 0.403) |
| FAC6 | -0.075 | (-0.626, 0.477) | 0.407 | (-0.170, 0.985) | 0.075 | (-0.451, 0.602) | 0.009 | (-0.553, 0.572) | -0.262 | (-0.083, 0.306) |

Values are given as mean and 95% confident interval (CI). FAC 1 (Omega 7 and 9 fatty acids and saturated fatty acids, total fatty acids, mono-unsaturated fatty acids), FAC2 (isoleucine, leucine, valine, phenylalanine, tyrosine and orosomucoid), FAC3 (acetate, alanine, lactate, pyruvate), FAC4 (esterified cholesterol, free cholesterol, omega 6 fatty acids, phosphoglycerides, phosphocholines and sphingomyelines), FAC5 (beta-hydroxybutyrate, citrate, histidine), FAC6 (acetoacetate, glutamine),

a p<0.05 from Q1, b p<0.05 from Q2, c p<0.05 from Q3, d p<0.05 from Q4
