# Supplementary material for: Adipose Tissue Dysfunction and Altered Systemic Amino Acid Metabolism Are Associated with Non-Alcoholic Fatty Liver Disease
Source: PLoS One. 2015 Oct 6;10(10):e0138889. doi: 10.1371/journal.pone.0138889 (PMC4595021; doi:10.1371/journal.pone.0138889)
Supplement: S3 Table — (DOCX) [file pone.0138889.s004.docx]

**Table S3.** Differentially expressed genes in the adipose tissue of the NAFL group.

| Genes | logFC | adj.p-value |
| --- | --- | --- |
| MMP9 | 2.60785 | 0.023017 |
| CHI3L1 | 2.48343 | 0.025925 |
| GRAPL | 1.50431 | 0.020513 |
| UCHL1 | 1.50431 | 0.020513 |
| TNMD | 1.38925 | 0.020513 |
| MXRA5 | 1.38622 | 0.01699 |
| MAP1B | 1.08333 | 0.014225 |
| GLIPR2 | 1.02031 | 0.023017 |
| WISP2 | 1.00201 | 0.022117 |
| BASP1 | 1.00011 | 0.033851 |
| SRPX2 | 0.94048 | 0.01699 |
| CD248 | 0.90631 | 0.01297 |
| APLNR | 0.853 | 0.046679 |
| VASN | 0.85021 | 0.034733 |
| FLNA | 0.82798 | 0.02927 |
| SLIT3 | 0.82564 | 0.028911 |
| PALLD | 0.82111 | 0.043214 |
| ABCC3 | 0.78433 | 0.028911 |
| NQO1 | 0.78025 | 0.042249 |
| ITGB5 | 0.77489 | 0.010211 |
| PLEKHO1 | 0.77315 | 0.043214 |
| MAN2B1 | 0.76415 | 0.018 |
| EXT1 | 0.75968 | 0.024213 |
| OLFML3 | 0.75265 | 0.010123 |
| SLC24A3 | 0.73954 | 0.043886 |
| GAA | 0.7379 | 0.044756 |
| KIAA1539 | 0.72983 | 0.043615 |
| C3 | 0.72426 | 0.017286 |
| TSHZ2 | 0.71198 | 0.021513 |
| COL6A2 | 0.70927 | 0.018673 |
| ENC1 | 0.70673 | 0.01699 |
| OLFML2B | 0.67935 | 0.01699 |
| PRICKLE1 | 0.66698 | 0.037256 |
| LAMB2 | 0.66619 | 0.016423 |
| CMTM3 | 0.66346 | 0.010123 |
| ITIH5 | 0.64825 | 0.010123 |
| RAB31 | 0.64749 | 0.03704 |
| BMP4 | 0.64057 | 0.023633 |
| FTH1 | 0.63913 | 0.023017 |
| PDLIM1 | 0.63477 | 0.010123 |
| DDX23 | 0.61437 | 0.01699 |
| VMO1 | 0.60673 | 0.034851 |
| HM13 | 0.60096 | 0.035984 |
| MAD1L1 | 0.59592 | 0.040829 |
| NA | 0.59582 | 0.024213 |
| COL16A1 | 0.595 | 0.01714 |
| C1orf54 | 0.58726 | 0.010123 |
| GLB1 | 0.58305 | 0.028008 |
| C11orf24 | 0.57773 | 0.042381 |
| NAGPA | 0.57712 | 0.035721 |
| RAD54L2 | 0.56452 | 0.045349 |
| FEM1A | 0.5617 | 0.043508 |
| SULF2 | 0.55286 | 0.048264 |
| PODN | 0.551 | 0.043844 |
| NA | 0.5472 | 0.039851 |
| C1R | 0.54455 | 0.028296 |
| TUBB2B | 0.54218 | 0.044645 |
| CSTA | 0.5384 | 0.043214 |
| PLCG2 | 0.53667 | 0.019103 |
| AOC2 | 0.53108 | 0.023017 |
| TUBB2A | 0.52665 | 0.044383 |
| LSP1 | 0.52219 | 0.049607 |
| HSPB7 | 0.52203 | 0.042381 |
| ABCC1 | 0.52117 | 0.015474 |
| GPC1 | 0.52056 | 0.024806 |
| TMEM45A | 0.52051 | 0.041149 |
| PRAF2 | 0.5175 | 0.011914 |
| RAB34 | 0.51202 | 0.019859 |
| GGT5 | 0.50883 | 0.016423 |
| CCDC80 | 0.50744 | 0.03533 |
| CUL9 | 0.50582 | 0.010123 |
| CLU | 0.50561 | 0.010123 |
| MANF | 0.50303 | 0.036404 |
| CCDC109B | 0.50288 | 0.042381 |
| C6orf145 | 0.50141 | 0.02122 |
| LAMB3 | 0.49913 | 0.033057 |
| SIL1 | 0.49912 | 0.033212 |
| KCND2 | 0.49904 | 0.048188 |
| ARF3 | 0.49674 | 0.010123 |
| MAN1B1 | 0.49636 | 0.021957 |
| ACTN1 | 0.49104 | 0.048421 |
| SIPA1L1 | 0.49013 | 0.024213 |
| LPCAT1 | 0.48663 | 0.041215 |
| GLG1 | 0.47841 | 0.031415 |
| ENO1 | 0.4779 | 0.036467 |
| INHBB | 0.4774 | 0.025807 |
| SYNGR2 | 0.47511 | 0.021957 |
| TBL3 | 0.47386 | 0.044645 |
| HOMER3 | 0.47275 | 0.023017 |
| COL6A1 | 0.47251 | 0.028699 |
| HTRA1 | 0.47129 | 0.042567 |
| PEA15 | 0.45864 | 0.016993 |
| SMPD1 | 0.45719 | 0.049551 |
| IFI27L2 | 0.45529 | 0.02927 |
| CTSA | 0.45417 | 0.034069 |
| GPR124 | 0.45375 | 0.03758 |
| CCDC137 | 0.45263 | 0.044516 |
| RPS6KA2 | 0.44728 | 0.023017 |
| PLEKHM2 | 0.44437 | 0.043435 |
| YWHAH | 0.44327 | 0.019463 |
| HMG20B | 0.43942 | 0.016471 |
| NA | 0.43933 | 0.03758 |
| TMEM219 | 0.43902 | 0.034069 |
| TMEM187 | 0.43843 | 0.01699 |
| CTBP2 | 0.43468 | 0.019581 |
| C1S | 0.43208 | 0.020513 |
| MFSD5 | 0.42305 | 0.04466 |
| TAX1BP3 | 0.42168 | 0.03758 |
| SUSD1 | 0.42132 | 0.020124 |
| PDLIM4 | 0.41899 | 0.01699 |
| HSPG2 | 0.41788 | 0.028606 |
| NUDT1 | 0.41706 | 0.019604 |
| FOXRED2 | 0.41577 | 0.03533 |
| USP32 | 0.41532 | 0.03758 |
| CALU | 0.41129 | 0.042249 |
| NAV1 | 0.41016 | 0.03533 |
| PI4K2A | 0.40733 | 0.033864 |
| DCLRE1B | 0.40599 | 0.044516 |
| NEK6 | 0.40391 | 0.01699 |
| QSOX1 | 0.40146 | 0.039189 |
| PFKP | 0.40032 | 0.03758 |
| UBE2D4 | 0.40024 | 0.032998 |
| SIDT2 | 0.39981 | 0.036118 |
| SCARB1 | 0.39969 | 0.04598 |
| ROR1 | 0.39839 | 0.01714 |
| MED22 | 0.39458 | 0.045487 |
| COG7 | 0.39401 | 0.049249 |
| CECR1 | 0.39136 | 0.020158 |
| FAM118B | 0.38776 | 0.043214 |
| CDR2L | 0.38609 | 0.01714 |
| ANXA11 | 0.38404 | 0.03533 |
| PTPN23 | 0.3826 | 0.01699 |
| PSAP | 0.37971 | 0.014225 |
| AP2B1 | 0.3789 | 0.016423 |
| C19orf61 | 0.37841 | 0.048148 |
| GNG2 | 0.37593 | 0.034725 |
| TRPC7 | 0.37488 | 0.028008 |
| C2orf42 | 0.37314 | 0.035965 |
| PAQR8 | 0.37223 | 0.03533 |
| TRIM38 | 0.37134 | 0.042181 |
| MAGEF1 | 0.36642 | 0.03758 |
| RPN1 | 0.36446 | 0.036118 |
| IER5L | 0.36392 | 0.034733 |
| FCGRT | 0.36355 | 0.021513 |
| TMEM127 | 0.35883 | 0.022117 |
| BRE | 0.35858 | 0.018608 |
| CHPF2 | 0.3554 | 0.047306 |
| JDP2 | 0.35269 | 0.022117 |
| CHRNE | 0.34826 | 0.01699 |
| GPSM2 | 0.34744 | 0.0486 |
| EIF2C1 | 0.34371 | 0.046442 |
| MEGF8 | 0.34063 | 0.02721 |
| C14orf174 | 0.33732 | 0.041149 |
| ADPRHL2 | 0.33605 | 0.024213 |
| DNAJC4 | 0.33329 | 0.035965 |
| NKX2-4 | 0.33286 | 0.01699 |
| RNF24 | 0.33282 | 0.034038 |
| SFXN3 | 0.33261 | 0.035965 |
| EHD4 | 0.33257 | 0.021957 |
| NA | 0.33241 | 0.036066 |
| CHD3 | 0.33051 | 0.01482 |
| TCTA | 0.3298 | 0.03758 |
| FAM129B | 0.32798 | 0.044234 |
| TULP4 | 0.32595 | 0.01699 |
| CREB3 | 0.32529 | 0.043886 |
| STAT3 | 0.32329 | 0.023017 |
| PPP1R9B | 0.32214 | 0.029167 |
| POLR2A | 0.32085 | 0.049425 |
| NA | 0.32032 | 0.01714 |
| SNCAIP | 0.31175 | 0.03593 |
| SCARB2 | 0.31033 | 0.030635 |
| SYNPO | 0.30993 | 0.044234 |
| CBARA1 | 0.30575 | 0.049901 |
| TTC7A | 0.30482 | 0.03758 |
| RAG2 | 0.3042 | 0.028911 |
| YKT6 | 0.30286 | 0.034069 |
| PDIA2 | 0.30115 | 0.043968 |
| S100G | 0.29993 | 0.03931 |
| PIGT | 0.29888 | 0.045698 |
| PCGF2 | 0.29844 | 0.038065 |
| PDGFA | 0.29764 | 0.044645 |
| SPANXN4 | 0.29753 | 0.038008 |
| S100A2 | 0.29753 | 0.042249 |
| EXO1 | 0.29597 | 0.018491 |
| NA | 0.29162 | 0.039189 |
| PSMD1 | 0.29112 | 0.043886 |
| AAGAB | 0.2897 | 0.039558 |
| C20orf71 | 0.28957 | 0.015565 |
| NA | 0.28754 | 0.043838 |
| VASH1 | 0.28581 | 0.03758 |
| LAMP1 | 0.28558 | 0.024213 |
| AKR1C2 | 0.28557 | 0.018465 |
| FBLIM1 | 0.28522 | 0.03533 |
| DACT1 | 0.28261 | 0.010123 |
| ZNF780B | 0.28091 | 0.044756 |
| CHST3 | 0.28025 | 0.043615 |
| VPS37C | 0.2801 | 0.028911 |
| SAMD4A | 0.27828 | 0.03779 |
| ZNF426 | 0.27697 | 0.044234 |
| PLEKHG2 | 0.27661 | 0.03533 |
| AKR1C1 | 0.27337 | 0.043214 |
| TRAPPC5 | 0.27187 | 0.030252 |
| KIAA0100 | 0.27104 | 0.034728 |
| PGK1 | 0.27025 | 0.036467 |
| DPAGT1 | 0.26993 | 0.042381 |
| MLPH | 0.26877 | 0.035051 |
| SPARC | 0.26744 | 0.043838 |
| RASA3 | 0.2658 | 0.049364 |
| FARP1 | 0.26436 | 0.048148 |
| ACADVL | 0.26369 | 0.043615 |
| AKR1C4 | 0.26192 | 0.044842 |
| NA | 0.26153 | 0.024832 |
| NFATC2 | 0.25514 | 0.042276 |
| LOXL2 | 0.25425 | 0.023017 |
| HIST1H2AM | 0.25331 | 0.045053 |
| NSFL1C | 0.25224 | 0.046442 |
| UGT2B17 | 0.25076 | 0.023017 |
| UBP1 | 0.25049 | 0.03229 |
| ACTR1A | 0.24984 | 0.03758 |
| SF4 | 0.24376 | 0.046442 |
| SLAMF7 | 0.24345 | 0.048253 |
| CARD6 | 0.24079 | 0.041132 |
| NUMBL | 0.23982 | 0.025807 |
| ZDHHC3 | 0.23547 | 0.024213 |
| ARID5A | 0.23263 | 0.041665 |
| TMED9 | 0.23255 | 0.01714 |
| DSN1 | 0.23151 | 0.043214 |
| CLPS | 0.22941 | 0.043214 |
| ECEL1 | 0.22863 | 0.043214 |
| RABIF | 0.22619 | 0.048306 |
| TMEM147 | 0.22562 | 0.049551 |
| PSMB3 | 0.22352 | 0.046072 |
| PAX2 | 0.2231 | 0.028467 |
| SLC9A1 | 0.22283 | 0.024804 |
| STOML1 | 0.22254 | 0.049901 |
| LIMD2 | 0.22143 | 0.029167 |
| TFEB | 0.22137 | 0.030588 |
| RUFY3 | 0.2199 | 0.022117 |
| C7orf59 | 0.21867 | 0.045487 |
| ZNF114 | 0.21819 | 0.047421 |
| FZD10 | 0.21644 | 0.038065 |
| C9orf169 | 0.21618 | 0.030168 |
| C14orf145 | 0.2157 | 0.029694 |
| EFCAB6 | 0.21425 | 0.043615 |
| TBR1 | 0.21229 | 0.048253 |
| KIF23 | 0.20935 | 0.039483 |
| DCAF8 | 0.20791 | 0.031623 |
| B4GALT5 | 0.20556 | 0.041807 |
| KIF3B | 0.20063 | 0.040149 |
| KRT71 | 0.19767 | 0.035715 |
| OSR1 | 0.19395 | 0.047879 |
| CYB561 | 0.19297 | 0.044645 |
| KIF3C | 0.1891 | 0.016423 |
| IL1F9 | 0.18226 | 0.04598 |
| TGFB3 | 0.18103 | 0.044645 |
| PAPLN | 0.17903 | 0.043214 |
| FGB | 0.17356 | 0.043214 |
| SLC39A10 | 0.17074 | 0.043615 |
| NALCN | 0.17047 | 0.01699 |
| PSMA4 | 0.16886 | 0.044645 |
| C11orf65 | 0.16553 | 0.048148 |
| CLRN1 | 0.15964 | 0.031251 |
| SYNPO2 | 0.1588 | 0.048306 |
| SPHKAP | 0.15849 | 0.044756 |
| ZNF483 | 0.14911 | 0.04671 |
| CDCA2 | 0.14457 | 0.048228 |
| BDNF | 0.14373 | 0.043214 |
| FBXL7 | 0.13988 | 0.019463 |
| ANK1 | 0.13868 | 0.048985 |
| TSPAN11 | 0.13643 | 0.045487 |
| TDRD1 | 0.13599 | 0.034069 |
| MTUS2 | 0.13455 | 0.048228 |
| WDR33 | -0.157517 | 0.034733 |
| UQCC | -0.161372 | 0.041415 |
| SEC11A | -0.167025 | 0.044516 |
| SEC11B | -0.167025 | 0.044516 |
| RPS25 | -0.169511 | 0.043886 |
| FXR1 | -0.180813 | 0.049427 |
| LRCH3 | -0.183115 | 0.023017 |
| GTF3C3 | -0.194378 | 0.034728 |
| TMX3 | -0.20702 | 0.016423 |
| ATF2 | -0.20939 | 0.021322 |
| TCEB1 | -0.210552 | 0.048306 |
| GCFC1 | -0.211019 | 0.043329 |
| EIF4H | -0.212912 | 0.039189 |
| BLVRB | -0.216593 | 0.042567 |
| TMEM50A | -0.217146 | 0.044756 |
| CRIPT | -0.22444 | 0.034728 |
| RPL11 | -0.225504 | 0.048253 |
| NAA30 | -0.229011 | 0.028162 |
| SPAG9 | -0.229064 | 0.033414 |
| PSMG2 | -0.229313 | 0.027544 |
| WDR90 | -0.230389 | 0.048421 |
| MRPL43 | -0.231001 | 0.028699 |
| MRPL15 | -0.232819 | 0.045487 |
| PIN1 | -0.234882 | 0.024213 |
| RPL30 | -0.235397 | 0.03758 |
| LRRIQ3 | -0.23616 | 0.044645 |
| EIF5 | -0.236393 | 0.043214 |
| MRPL36 | -0.239362 | 0.016471 |
| ASNSD1 | -0.239986 | 0.03533 |
| CCDC56 | -0.2401 | 0.028008 |
| MAPK14 | -0.240746 | 0.026636 |
| CCDC50 | -0.242366 | 0.049427 |
| NDUFA12 | -0.244142 | 0.022456 |
| IPO7 | -0.246694 | 0.041634 |
| SEH1L | -0.248379 | 0.037969 |
| TARS | -0.248676 | 0.035732 |
| NARG2 | -0.248705 | 0.018 |
| LGALS3 | -0.249926 | 0.043383 |
| PRKAG1 | -0.250975 | 0.043214 |
| ZNF331 | -0.253308 | 0.046819 |
| STAU2 | -0.253344 | 0.024806 |
| CDC5L | -0.25511 | 0.045053 |
| ALDH7A1 | -0.255935 | 0.016923 |
| DAP3 | -0.258863 | 0.022809 |
| ARIH1 | -0.260824 | 0.045051 |
| ANKRD46 | -0.262204 | 0.039483 |
| DDX47 | -0.264762 | 0.043214 |
| NA | -0.268273 | 0.042181 |
| TRIM56 | -0.269837 | 0.03533 |
| MRPL16 | -0.271235 | 0.042381 |
| ZC3H15 | -0.273625 | 0.01297 |
| C10orf57 | -0.273965 | 0.02496 |
| C10orf58 | -0.273965 | 0.02496 |
| SERBP1 | -0.274174 | 0.025123 |
| C7orf30 | -0.274845 | 0.034038 |
| C22orf32 | -0.275079 | 0.042381 |
| TMEM126B | -0.276146 | 0.03758 |
| USP8 | -0.276797 | 0.029167 |
| RFC1 | -0.276816 | 0.036404 |
| ELF2 | -0.278072 | 0.043329 |
| PPM1A | -0.278376 | 0.037256 |
| OXNAD1 | -0.278937 | 0.028008 |
| MIB1 | -0.280017 | 0.045053 |
| SECISBP2 | -0.282693 | 0.03533 |
| POLR2D | -0.283379 | 0.025848 |
| LONP2 | -0.2836 | 0.022117 |
| PAPOLA | -0.284671 | 0.046442 |
| NAMPT | -0.28477 | 0.029432 |
| PSMB7 | -0.28583 | 0.01699 |
| SLC1A3 | -0.291874 | 0.024213 |
| NA | -0.292863 | 0.030761 |
| VPS37A | -0.292868 | 0.028173 |
| GNPAT | -0.295263 | 0.01699 |
| NDUFB6 | -0.295897 | 0.021513 |
| HNRNPA1 | -0.298517 | 0.03533 |
| HNRPA1L3 | -0.298517 | 0.03533 |
| RPS6 | -0.298694 | 0.023017 |
| TMEM106B | -0.299528 | 0.036095 |
| TIAL1 | -0.300882 | 0.02349 |
| SHPRH | -0.303283 | 0.02783 |
| L2HGDH | -0.303685 | 0.024038 |
| KLHL8 | -0.305511 | 0.03533 |
| MRPL33 | -0.305962 | 0.034728 |
| PPP2R5A | -0.306448 | 0.033851 |
| RBM18 | -0.308144 | 0.045053 |
| VEZT | -0.311388 | 0.042101 |
| RAP1GDS1 | -0.313423 | 0.023017 |
| ERCC8 | -0.313951 | 0.033212 |
| ATP6V1C1 | -0.315962 | 0.03553 |
| RPL8 | -0.316193 | 0.024038 |
| HNRNPD | -0.317673 | 0.043838 |
| DDHD2 | -0.31773 | 0.014202 |
| XRN2 | -0.318076 | 0.043617 |
| PDPK1 | -0.318719 | 0.049841 |
| TBCK | -0.319423 | 0.047421 |
| DSCR3 | -0.319517 | 0.03758 |
| L3MBTL4 | -0.32023 | 0.045051 |
| OXA1L | -0.320738 | 0.024213 |
| COX5A | -0.321013 | 0.033246 |
| MRPS22 | -0.321053 | 0.036066 |
| GALK2 | -0.321417 | 0.019463 |
| C6orf120 | -0.321779 | 0.047408 |
| LPIN2 | -0.322342 | 0.034354 |
| LENG1 | -0.323143 | 0.023017 |
| GART | -0.323442 | 0.017116 |
| PRKRA | -0.323979 | 0.029694 |
| PRKRAP1 | -0.323979 | 0.029694 |
| GHITM | -0.32505 | 0.048985 |
| PHB2 | -0.325074 | 0.01699 |
| RPS6KB1 | -0.325624 | 0.031623 |
| IMMP2L | -0.327061 | 0.046442 |
| FAM200A | -0.32896 | 0.03533 |
| CHCHD7 | -0.32947 | 0.022117 |
| REV1 | -0.33031 | 0.03758 |
| TM7SF3 | -0.330384 | 0.010211 |
| PLEKHF2 | -0.330501 | 0.048228 |
| PTER | -0.332149 | 0.03758 |
| RNF111 | -0.333016 | 0.042567 |
| GOLGA4 | -0.334782 | 0.03779 |
| GAS1 | -0.335222 | 0.021169 |
| MRPL45 | -0.336866 | 0.02122 |
| NAA20 | -0.337475 | 0.01699 |
| NOL7 | -0.337848 | 0.033099 |
| YIPF4 | -0.34055 | 0.020658 |
| TSPAN2 | -0.340738 | 0.044516 |
| TRAPPC8 | -0.341055 | 0.042381 |
| ISCA2 | -0.341614 | 0.022642 |
| GNL2 | -0.341771 | 0.024213 |
| STYX | -0.342518 | 0.019254 |
| SYF2 | -0.342866 | 0.043329 |
| SP3 | -0.343781 | 0.041665 |
| MOBKL3 | -0.344366 | 0.030757 |
| EIF1 | -0.345172 | 0.016923 |
| ANKRD13C | -0.346271 | 0.023017 |
| DPY19L4 | -0.346348 | 0.036251 |
| SLTM | -0.34726 | 0.028911 |
| EGLN1 | -0.347274 | 0.043214 |
| MRPL46 | -0.347855 | 0.030497 |
| REXO2 | -0.349145 | 0.045487 |
| KLHL9 | -0.350704 | 0.043886 |
| AIMP1 | -0.350747 | 0.028173 |
| ARHGAP5 | -0.350899 | 0.022117 |
| PRKAR1A | -0.350972 | 0.01699 |
| CCT4 | -0.351025 | 0.049901 |
| PHYH | -0.351079 | 0.019581 |
| GATAD1 | -0.35127 | 0.044516 |
| LIAS | -0.353196 | 0.043753 |
| TMEM167A | -0.354226 | 0.035956 |
| ACADSB | -0.35469 | 0.039674 |
| AGTPBP1 | -0.355941 | 0.041682 |
| RMND1 | -0.356143 | 0.024113 |
| CYC1 | -0.35839 | 0.018 |
| RPAP2 | -0.35947 | 0.03758 |
| TMEM14C | -0.362269 | 0.023258 |
| SS18 | -0.362957 | 0.023017 |
| LRRC8C | -0.3636 | 0.045487 |
| CHMP5 | -0.36398 | 0.024213 |
| RPS12 | -0.365842 | 0.043753 |
| MTHFD1 | -0.365905 | 0.023017 |
| SH3GLB1 | -0.365929 | 0.019587 |
| GLO1 | -0.365951 | 0.048148 |
| LRP11 | -0.36803 | 0.03931 |
| AZI2 | -0.368197 | 0.01699 |
| TTC21B | -0.368417 | 0.023017 |
| SLC25A22 | -0.368479 | 0.038551 |
| TOR1AIP1 | -0.369611 | 0.013643 |
| PDHB | -0.370267 | 0.033282 |
| NDUFS4 | -0.370927 | 0.015565 |
| DHRS7 | -0.372505 | 0.028687 |
| HSDL2 | -0.373672 | 0.01699 |
| TSPAN3 | -0.374218 | 0.034069 |
| NDUFAF4 | -0.374791 | 0.03758 |
| ZCCHC8 | -0.374808 | 0.023017 |
| ATP5L | -0.3767 | 0.024213 |
| PCDH18 | -0.377423 | 0.026432 |
| CLDND1 | -0.377726 | 0.024213 |
| PRDX6 | -0.378273 | 0.01699 |
| HIVEP2 | -0.37844 | 0.022117 |
| RANBP9 | -0.381224 | 0.022456 |
| CALM2 | -0.38182 | 0.028162 |
| FAM188A | -0.382319 | 0.029167 |
| COPS2 | -0.382681 | 0.043838 |
| MITD1 | -0.383264 | 0.043214 |
| FAM179B | -0.384032 | 0.043214 |
| SNRNP27 | -0.384338 | 0.044234 |
| OGT | -0.385909 | 0.043617 |
| MRPL32 | -0.387204 | 0.011182 |
| ALDH9A1 | -0.387877 | 0.048188 |
| MRPS31 | -0.388526 | 0.023017 |
| ACP1 | -0.388742 | 0.017286 |
| ARRDC3 | -0.389373 | 0.034889 |
| NCRNA00188 | -0.389958 | 0.038551 |
| CRLS1 | -0.389994 | 0.019581 |
| EIF2A | -0.39061 | 0.01699 |
| RABGGTB | -0.391111 | 0.023017 |
| SYAP1 | -0.391278 | 0.02721 |
| BAG2 | -0.391618 | 0.03002 |
| FUBP1 | -0.391666 | 0.043214 |
| ALG13 | -0.391778 | 0.038065 |
| APPBP2 | -0.391899 | 0.04598 |
| ZBTB25 | -0.394614 | 0.022117 |
| SBF2 | -0.395112 | 0.028759 |
| SUCLG2 | -0.396784 | 0.023633 |
| RHOT1 | -0.398503 | 0.029167 |
| ORC3L | -0.398534 | 0.028911 |
| PCCA | -0.400012 | 0.019463 |
| PHAX | -0.400642 | 0.028162 |
| SNX3 | -0.40194 | 0.018 |
| ACTR10 | -0.402279 | 0.029167 |
| TAF9B | -0.406265 | 0.045487 |
| METTL5 | -0.407252 | 0.016471 |
| SCML1 | -0.409061 | 0.034069 |
| C1orf43 | -0.410491 | 0.021513 |
| PRPSAP2 | -0.411918 | 0.020949 |
| CCNH | -0.413611 | 0.025157 |
| FERMT2 | -0.413838 | 0.02312 |
| NUP54 | -0.414589 | 0.031936 |
| DCTN6 | -0.414957 | 0.038604 |
| NR3C1 | -0.41499 | 0.032679 |
| MED21 | -0.417096 | 0.04466 |
| EIF3M | -0.418784 | 0.033246 |
| POLR3E | -0.418865 | 0.025399 |
| LPL | -0.419424 | 0.01699 |
| C2orf64 | -0.41975 | 0.044645 |
| CAPN7 | -0.42-0637 | 0.047686 |
| CGGBP1 | -0.421368 | 0.01699 |
| DCXR | -0.421702 | 0.028008 |
| DHRS3 | -0.421927 | 0.022842 |
| TRAPPC3 | -0.422834 | 0.023017 |
| ZDHHC2 | -0.430131 | 0.039851 |
| PPID | -0.430219 | 0.02721 |
| NUP37 | -0.430562 | 0.031936 |
| KDM6A | -0.430577 | 0.039851 |
| FH | -0.434211 | 0.010123 |
| SLC25A4 | -0.435674 | 0.019581 |
| MRPS10 | -0.435853 | 0.01699 |
| TXNDC16 | -0.436286 | 0.03758 |
| DDIT3 | -0.442421 | 0.045487 |
| DLD | -0.443176 | 0.019859 |
| ZNF627 | -0.444123 | 0.042381 |
| USP13 | -0.444175 | 0.024213 |
| PJA1 | -0.444732 | 0.039512 |
| ANO6 | -0.445109 | 0.010123 |
| PTP4A1 | -0.44576 | 0.049425 |
| COQ5 | -0.446026 | 0.022117 |
| RPL36AL | -0.446863 | 0.022456 |
| NA | -0.447224 | 0.044234 |
| RAD21 | -0.453383 | 0.044234 |
| PTPN13 | -0.453989 | 0.043214 |
| SUCLG1 | -0.454522 | 0.010211 |
| UQCRC2 | -0.454543 | 0.043329 |
| CRBN | -0.455398 | 0.022117 |
| NFU1 | -0.456022 | 0.010123 |
| CCT2 | -0.457253 | 0.018 |
| NDUFA10 | -0.458674 | 0.01699 |
| BRIX1 | -0.459688 | 0.044756 |
| GHR | -0.46167 | 0.010123 |
| NA | -0.462759 | 0.045487 |
| BTF3 | -0.466131 | 0.022456 |
| PGRMC1 | -0.466435 | 0.034069 |
| NA | -0.46747 | 0.032397 |
| WRB | -0.468005 | 0.030246 |
| PFDN2 | -0.472016 | 0.028911 |
| SCAI | -0.472073 | 0.036415 |
| LPHN2 | -0.473057 | 0.044234 |
| TMEM50B | -0.476061 | 0.043753 |
| MTERFD3 | -0.477121 | 0.044234 |
| CYBRD1 | -0.477183 | 0.048306 |
| DNAJC19 | -0.479489 | 0.011914 |
| MGEA5 | -0.48092 | 0.024213 |
| KIAA0776 | -0.481404 | 0.04613 |
| CYP20A1 | -0.483767 | 0.01699 |
| SFT2D1 | -0.483976 | 0.043886 |
| C3orf23 | -0.484029 | 0.022117 |
| TXNDC12 | -0.485421 | 0.024213 |
| XBP1 | -0.48554 | 0.024213 |
| USP25 | -0.485967 | 0.01297 |
| PCGF6 | -0.486509 | 0.02349 |
| CHD1 | -0.48722 | 0.043214 |
| MRS2 | -0.487983 | 0.030246 |
| ELOVL5 | -0.488878 | 0.043214 |
| ATP5S | -0.48965 | 0.010777 |
| CHKA | -0.491112 | 0.026249 |
| UBA2 | -0.492862 | 0.036118 |
| NACA | -0.493821 | 0.010123 |
| NACA2 | -0.493821 | 0.010123 |
| NACAP1 | -0.493821 | 0.010123 |
| NA | -0.494851 | 0.016423 |
| PPP3R1 | -0.497951 | 0.039512 |
| CEPT1 | -0.500092 | 0.01714 |
| CD302 | -0.500766 | 0.021703 |
| VEGFA | -0.501087 | 0.024806 |
| FBXL5 | -0.502317 | 0.023017 |
| YARS2 | -0.503254 | 0.045051 |
| SLC25A6 | -0.505256 | 0.011182 |
| BCKDHB | -0.505368 | 0.023017 |
| PGM1 | -0.505579 | 0.020513 |
| C6orf72 | -0.505844 | 0.024213 |
| ADIPOQ | -0.505917 | 0.023017 |
| ARID4A | -0.50613 | 0.028911 |
| ABHD15 | -0.506363 | 0.023017 |
| GADD45A | -0.508105 | 0.03365 |
| KIAA1737 | -0.508135 | 0.031449 |
| ABCC9 | -0.508279 | 0.024044 |
| NDFIP2 | -0.509692 | 0.031446 |
| PDE8A | -0.510835 | 0.028008 |
| MOSC2 | -0.5127 | 0.024213 |
| UBE2W | -0.51914 | 0.011914 |
| ZNF791 | -0.519868 | 0.023017 |
| SLC25A16 | -0.523374 | 0.022117 |
| MNAT1 | -0.527825 | 0.028162 |
| ACTR6 | -0.528217 | 0.023017 |
| CYB5A | -0.529388 | 0.028008 |
| C18orf21 | -0.529861 | 0.023633 |
| HADH | -0.5299 | 0.010123 |
| PCBD1 | -0.530288 | 0.015967 |
| OXCT1 | -0.533995 | 0.049551 |
| CHORDC1 | -0.536691 | 0.033727 |
| PRDX3 | -0.537081 | 0.043214 |
| TIGD2 | -0.537451 | 0.022456 |
| C17orf58 | -0.541113 | 0.045051 |
| PAPSS1 | -0.543791 | 0.010211 |
| NET1 | -0.544566 | 0.048594 |
| PKN2 | -0.546394 | 0.01699 |
| SCOC | -0.546506 | 0.03758 |
| LPIN1 | -0.548224 | 0.040506 |
| ATAD1 | -0.55044 | 0.045487 |
| GLUL | -0.55154 | 0.01297 |
| FAM89A | -0.551643 | 0.0232 |
| EFHA2 | -0.552081 | 0.018 |
| ADH1B | -0.552458 | 0.029776 |
| PGAM5 | -0.55364 | 0.025157 |
| PXMP2 | -0.55364 | 0.025157 |
| PMM1 | -0.555401 | 0.033851 |
| PDHX | -0.555487 | 0.01699 |
| EPB41L2 | -0.556467 | 0.024213 |
| RIOK3 | -0.558565 | 0.035586 |
| HNRNPH3 | -0.561568 | 0.022809 |
| MID2 | -0.56178 | 0.016923 |
| RTCD1 | -0.562869 | 0.015565 |
| RBMX | -0.563848 | 0.03533 |
| PTGES3 | -0.565172 | 0.049551 |
| ACVR1C | -0.569771 | 0.012217 |
| SCO1 | -0.571585 | 0.033851 |
| TJP2 | -0.5716-01 | 0.01482 |
| NUDT6 | -0.571882 | 0.043838 |
| ADH5 | -0.571936 | 0.037781 |
| TMEM66 | -0.572725 | 0.035457 |
| PAIP2 | -0.573024 | 0.038065 |
| NDUFB5 | -0.580037 | 0.026636 |
| ACSL1 | -0.583271 | 0.02783 |
| NDUFS1 | -0.585922 | 0.023017 |
| ISOC1 | -0.587447 | 0.042381 |
| HSCB | -0.588405 | 0.018 |
| ZFAND5 | -0.589267 | 0.033057 |
| F3 | -0.589944 | 0.044645 |
| NA | -0.590225 | 0.029167 |
| LSM6 | -0.591193 | 0.010123 |
| MRPL44 | -0.594162 | 0.024213 |
| CASD1 | -0.597757 | 0.021322 |
| FAM10A4 | -0.601858 | 0.022958 |
| ST13 | -0.601858 | 0.022958 |
| NRIP1 | -0.606777 | 0.010123 |
| BNIP3L | -0.607466 | 0.01699 |
| SDHB | -0.608167 | 0.015041 |
| ZNF143 | -0.609619 | 0.036551 |
| PIGP | -0.61309 | 0.018542 |
| KLF15 | -0.613782 | 0.024806 |
| BRP44L | -0.613836 | 0.024649 |
| MPDZ | -0.619762 | 0.026158 |
| GPD1L | -0.619932 | 0.021513 |
| CDKN1B | -0.627122 | 0.033038 |
| ACADM | -0.631125 | 0.01699 |
| MORC3 | -0.635374 | 0.044234 |
| BTNL9 | -0.636858 | 0.042381 |
| ACSS3 | -0.640457 | 0.048253 |
| CETN3 | -0.640712 | 0.027008 |
| ALPK3 | -0.642259 | 0.028162 |
| APIP | -0.642621 | 0.034038 |
| NMD3 | -0.643574 | 0.047686 |
| MAPK10 | -0.645391 | 0.029167 |
| TMEM69 | -0.64601 | 0.043214 |
| CSAD | -0.646023 | 0.011182 |
| SGCG | -0.646886 | 0.024806 |
| PECR | -0.650048 | 0.023017 |
| TGDS | -0.65115 | 0.024213 |
| NUP133 | -0.651338 | 0.039189 |
| OXR1 | -0.653814 | 0.029776 |
| GNAI1 | -0.656239 | 0.043615 |
| LYRM5 | -0.65968 | 0.044756 |
| GPHN | -0.660395 | 0.03745 |
| MRPL39 | -0.665689 | 0.010123 |
| TWIST1 | -0.66732 | 0.01297 |
| HIBADH | -0.669165 | 0.01699 |
| MATN2 | -0.670196 | 0.023017 |
| CXCR7 | -0.674695 | 0.039851 |
| TMEM135 | -0.68147 | 0.017092 |
| PFKFB3 | -0.686116 | 0.022117 |
| C6orf211 | -0.687141 | 0.048188 |
| NRBF2 | -0.688001 | 0.01297 |
| TNFAIP8 | -0.688762 | 0.033864 |
| ECHDC3 | -0.688883 | 0.010211 |
| LRIG1 | -0.689571 | 0.014225 |
| AQP7 | -0.689598 | 0.022117 |
| MCEE | -0.693642 | 0.033047 |
| ABHD3 | -0.694039 | 0.023017 |
| RTN3 | -0.694501 | 0.01699 |
| ANKRA2 | -0.694595 | 0.028699 |
| RGS3 | -0.694891 | 0.028165 |
| EIF1B | -0.696905 | 0.01699 |
| NA | -0.699059 | 0.035956 |
| AASS | -0.706357 | 0.022456 |
| IGF2BP2 | -0.707997 | 0.048228 |
| NIPSNAP3A | -0.70935 | 0.04343 |
| CNBP | -0.714437 | 0.01699 |
| CMC1 | -0.722366 | 0.019581 |
| HNRPDL | -0.725536 | 0.022523 |
| ZFYVE21 | -0.72678 | 0.018491 |
| KIAA1370 | -0.727427 | 0.043214 |
| IDH1 | -0.729478 | 0.03758 |
| SEC24B | -0.730134 | 0.033851 |
| BMPR1A | -0.737703 | 0.041665 |
| RASL10B | -0.739567 | 0.039512 |
| AUH | -0.744403 | 0.022203 |
| RDH10 | -0.747339 | 0.03779 |
| NR1D2 | -0.755695 | 0.032385 |
| CS | -0.760634 | 0.047686 |
| ZFAND1 | -0.773173 | 0.036095 |
| NA | -0.775371 | 0.010123 |
| PPP1R16A | -0.790061 | 0.016423 |
| HEPN1 | -0.792223 | 0.034354 |
| HRSP12 | -0.801461 | 0.010123 |
| HMGB2 | -0.802825 | 0.01699 |
| TMEM100 | -0.803002 | 0.024213 |
| MRPL19 | -0.806882 | 0.023017 |
| PRKAR2B | -0.815576 | 0.022523 |
| GPN3 | -0.823437 | 0.022523 |
| ST3GAL6 | -0.823456 | 0.029432 |
| CDKN2C | -0.833027 | 0.01699 |
| TMEM22 | -0.84662 | 0.010123 |
| VBP1 | -0.853517 | 0.028324 |
| SERPINI1 | -0.876133 | 0.03758 |
| SLC19A3 | -0.87785 | 0.022523 |
| SULF1 | -0.880902 | 0.043214 |
| ATP5A1 | -0.882827 | 0.025807 |
| ANXA3 | -0.885051 | 0.043214 |
| PTGR1 | -0.88633 | 0.03758 |
| CALCRL | -0.90304 | 0.018 |
| CMTM8 | -0.93633 | 0.02808 |
| FGFBP2 | -0.940525 | 0.047421 |
| GPR160 | -0.961768 | 0.035965 |
| LACTB2 | -0.967917 | 0.010123 |
| ETFA | -0.982802 | 0.010123 |
| SUCLA2 | -0.984477 | 0.028008 |
| STOX1 | -1.018035 | 0.028162 |
| EGFLAM | -1.067201 | 0.022523 |
| ABHD5 | -1.101775 | 0.01699 |
| CASQ2 | -1.206044 | 0.022456 |
| AZGP1 | -1.470061 | 0.035457 |
| DEFB132 | -1.520434 | 0.024806 |
| AGPAT9 | -1.734021 | 0.038799 |
| CA3 | -1.777514 | 0.029167 |
| C12orf39 | -2.779092 | 0.010123 |
