# Supplementary material for: Adipose Tissue Dysfunction and Altered Systemic Amino Acid Metabolism Are Associated with Non-Alcoholic Fatty Liver Disease
Source: PLoS One. 2015 Oct 6;10(10):e0138889. doi: 10.1371/journal.pone.0138889 (PMC4595021; doi:10.1371/journal.pone.0138889)
Supplement: S4 Table — (DOCX) [file pone.0138889.s005.docx]

S4 Table. Abbreviations and full names of metabolites

Abbreviation Full name Unit

| Alb | Albumin | signal area |
| --- | --- | --- |
| XXL_VLDL_PL | Phospholipids in chylomicrons and extremely large VLDL | mmol/l |
| XXL_VLDL_L | Total lipids in chylomicrons and extremely large VLDL | mmol/l |
| XXL_VLDL_P | Concentration of chylomicrons and extremely large VLDL particles | mol/l |
| XL_VLDL_PL | Phospholipids in very large VLDL | mmol/l |
| XL_VLDL_TG | Triglycerides in very large VLDL | mmol/l |
| XL_VLDL_L | Total lipids in very large VLDL | mmol/l |
| XL_VLDL_P | Concentration of very large VLDL particles | mol/l |
| L_VLDL_C | Total cholesterol in large VLDL | mmol/l |
| L_VLDL_FC | Free cholesterol in large VLDL | mmol/l |
| L_VLDL_PL | Phospholipids in large VLDL | mmol/l |
| L_VLDL_TG | Triglycerides in large VLDL | mmol/l |
| L_VLDL_CE | Cholesterol esters in large VLDL | mmol/l |
| L_VLDL_L | Total lipids in large VLDL | mmol/l |
| L_VLDL_P | Concentration of large VLDL particles | mol/l |
| M_VLDL_C | Total cholesterol in medium VLDL | mmol/l |
| M_VLDL_FC | Free cholesterol in medium VLDL | mmol/l |
| M_VLDL_PL | Phospholipids in medium VLDL | mmol/l |
| M_VLDL_TG | Triglycerides in medium VLDL | mmol/l |
| M_VLDL_CE | Cholesterol esters in medium VLDL | mmol/l |
| M_VLDL_L | Total lipids in medium VLDL | mmol/l |
| M_VLDL_P | Concentration of medium VLDL particles | mol/l |
| S_VLDL_C | Total cholesterol in small VLDL | mmol/l |
| S_VLDL_FC | Free cholesterol in small VLDL | mmol/l |
| S_VLDL_PL | Phospholipids in small VLDL | mmol/l |
| S_VLDL_TG | Triglycerides in small VLDL | mmol/l |
| S_VLDL_L | Total lipids in small VLDL | mmol/l |
| S_VLDL_P | Concentration of small VLDL particles | mol/l |
| XS_VLDL_PL | Phospholipids in very small VLDL | mmol/l |
| XS_VLDL_TG | Triglycerides in very small VLDL | mmol/l |
| XS_VLDL_L | Total lipids in very small VLDL | mmol/l |
| XS_VLDL_P | Concentration of very small VLDL particles | mol/l |
| IDL_FC | Free cholesterol in IDL | mmol/l |
| IDL_PL | Phospholipids in IDL | mmol/l |
| IDL_L | Total lipids in IDL | mmol/l |
| IDL_P | Concentration of IDL particles | mol/l |
| L_LDL_C | Total cholesterol in large LDL | mmol/l |
| L_LDL_FC | Free cholesterol in large LDL | mmol/l |
| L_LDL_PL | Phospholipids in large LDL | mmol/l |
| L_LDL_CE | Cholesterol esters in large LDL | mmol/l |
| L_LDL_L | Total lipids in large LDL | mmol/l |
| L_LDL_P | Concentration of large LDL particles | mol/l |
| M_LDL_C | Total cholesterol in medium LDL | mmol/l |
| M_LDL_PL | Phospholipids in medium LDL | mmol/l |
| M_LDL_CE | Cholesterol esters in medium LDL | mmol/l |
| M_LDL_L | Total lipids in medium LDL | mmol/l |
| M_LDL_P | Concentration of medium LDL particles | mol/l |
| S_LDL_C | Total cholesterol in small LDL | mmol/l |
| S_LDL_L | Total lipids in small LDL | mmol/l |
| S_LDL_P | Concentration of small LDL particles | mol/l |
| XL_HDL_C | Total cholesterol in very large HDL | mmol/l |
| XL_HDL_FC | Free cholesterol in very large HDL | mmol/l |
| XL_HDL_PL | Phospholipids in very large HDL | mmol/l |
| XL_HDL_TG | Triglycerides in very large HDL | mmol/l |
| XL_HDL_CE | Cholesterol esters in very large HDL | mmol/l |
| XL_HDL_L | Total lipids in very large HDL | mmol/l |
| XL_HDL_P | Concentration of very large HDL particles | mol/l |
| L_HDL_C | Total cholesterol in large HDL | mmol/l |
| L_HDL_FC | Free cholesterol in large HDL | mmol/l |
| L_HDL_PL | Phospholipids in large HDL | mmol/l |
| L_HDL_CE | Cholesterol esters in large HDL | mmol/l |
| L_HDL_L | Total lipids in large HDL | mmol/l |
| L_HDL_P | Concentration of large HDL particles | mol/l |
| M_HDL_C | Total cholesterol in medium HDL | mmol/l |
| M_HDL_FC | Free cholesterol in medium HDL | mmol/l |
| M_HDL_PL | Phospholipids in medium HDL | mmol/l |
| M_HDL_CE | Cholesterol esters in medium HDL | mmol/l |
| M_HDL_L | Total lipids in medium HDL | mmol/l |
| M_HDL_P | Concentration of medium HDL particles | mol/l |
| S_HDL_TG | Triglycerides in small HDL | mmol/l |
| S_HDL_L | Total lipids in small HDL | mmol/l |
| S_HDL_P | Concentration of small HDL particles | mol/l |
| XXL_VLDL_TG | Triglycerides in chylomicrons and extremely large VLDL | mmol/l |
| VLDL_TG | Triglycerides in VLDL | mmol/l |
| IDL_TG | Triglycerides in IDL | mmol/l |
| IDL_C | Total cholesterol in IDL | mmol/l |
| LDL_C | Total cholesterol in LDL | mmol/l |
| HDL_C | Total cholesterol in HDL | mmol/l |
| Serum_TG | Serum total triglycerides | mmol/l |
| Serum_C | Serum total cholesterol | mmol/l |
| VLDL_D | Mean diameter for VLDL particles | nm |
| LDL_D | Mean diameter for LDL particles | nm |
| HDL_D | Mean diameter for HDL particles | nm |
| VLDL_TG_eFR | Triglycerides in VLDL * | mmol/l |
| IDL_C_eFR | Total cholesterol in IDL * | mmol/l |
| LDL_C_eFR | Total cholesterol in LDL * | mmol/l |
| HDL2_C | Total cholesterol in HDL2 * | mmol/l |
| ApoA1 | Apolipoprotein A-I * | g/l |
| ApoB | Apolipoprotein B * | g/l |
| ApoBtoApoA1 | Apolipoprotein B by apolipoprotein A-I * |  |
| HDL3_C | Total cholesterol in HDL3 * | mmol/l |
| bOHBut | 3-hydroxybutyrate | mmol/l |
| Ace | Acetate | mmol/l |
| AcAce | Acetoacetate | mmol/l |
| Ala | Alanine | mmol/l |
| MobCH2 | CH2 groups of mobile lipids | mmol/l |
| MobCH3 | CH3 groups of mobile lipids | mmol/l |
| Cit | Citrate | mmol/l |
| Crea | Creatinine | mmol/l |
| MobCH | Double bond protons of mobile lipids | mmol/l |
| Glc | Glucose | mmol/l |
| Gln | Glutamine | mmol/l |
| Glol | Glycerol | mmol/l |
| Gly | Glycine | mmol/l |
| Gp | Glycoprotein acetyls, mainly a1-acid glycoprotein | mmol/l |
| His | Histidine | mmol/l |
| Ile | Isoleucine | mmol/l |
| Lac | Lactate | mmol/l |
| Leu | Leucine | mmol/l |
| Phe | Phenylalanine | mmol/l |
| Pyr | Pyruvate | mmol/l |
| Tyr | Tyrosine | mmol/l |
| Urea | Urea | mmol/l |
| Val | Valine | mmol/l |
| EstC | Esterified cholesterol | mmol/l |
| FreeC | Free cholesterol | mmol/l |
| FAw3 | Omega-3 fatty acids | mmol/l |
| FAw6 | Omega-6 fatty acids | mmol/l |
| FAw79S | Omega-7, omega-9 and saturated fatty acids | mmol/l |
| TotFA | Total fatty acids | mmol/l |
| LA | 18:2, linoleic acid | mmol/l |
| otPUFA | Other polyunsaturated fatty acids than 18:2 | ** |
| DHA | 22:6, docosahexaenoic acid | mmol/l |
| MUFA | Monounsaturated fatty acids; 16:1, 18:1 | mmol/l |
| TotPG | Total phosphoglycerides | mmol/l |
| PC | Phosphatidylcholine and other cholines | mmol/l |
| SM | Sphingomyelins | mmol/l |
| FAw3toFA | Ratio of omega-3 fatty acids to total fatty acids | % |
| FAw6toFA | Ratio of omega-6 fatty acids to total fatty acids | % |
| FAw79StoFA | Ratio of omega-7, omega-9 and saturated fatty acids to total fatty acids | % |
| CH2inFA | Average number of methylene groups in a fatty acid chain |  |
| TGtoPG | Ratio of triglycerides to phosphoglycerides |  |
| CH2toDB | Average number of methylene groups per a double bond |  |
| DBinFA | Average number of double bonds in a fatty acid chain |  |
| BIStoDB | Ratio of bisallylic groups to double bonds |  |
| BIStoFA | Ratio of bisallylic groups to total fatty acids |  |
| FALen | Description of average fatty acid chain length, not actual carbon number |  |

*Values estimated with the Extended Friedewald method.
